# Determinants of Cat Choice and Outcomes for Adult Cats and Kittens Adopted from an Australian Animal Shelter

**DOI:** 10.3390/ani5020276

**Published:** 2015-04-29

**Authors:** Sarah Zito, Mandy Paterson, Dianne Vankan, John Morton, Pauleen Bennett, Clive Phillips

**Affiliations:** 1 Centre for Animal Welfare and Ethics, School of Veterinary Science, University of Queensland, Gatton Campus, Gatton, QLD 4343, Australia; 2 RSPCA Queensland, Locked Bag 3000, Archerfield BH, QLD 4108, Australia; E-Mail: mpaterson@rspcaqld.org.au; 3 School of Veterinary Science, University of Queensland, Gatton Campus, Gatton, QLD 4343, Australia; E-Mails: dianne.stephens@outlook.com (D.V.); john.morton@optusnet.com.au (J.M.); 4 School of Psychology and Public Health, La Trobe University, Bendigo, VIC 3552, Australia; E-Mail: Pauleen.Bennett@latrobe.edu.au

**Keywords:** adult cat and kitten adoption, cat choice, low-cost adoption, animal shelter, unwanted cats, shelter medicine, cat adoption outcomes

## Abstract

**Simple Summary:**

Commonly, more adult cats than kittens are euthanized in animal shelters. We surveyed 382 cat adopters to assess adoption outcomes and potential determinants of adopters’ choice of cat age group and price. Most adopters had benevolent motivations for adopting from the shelter and had put considerable thought into the adoption and responsible ownership requirements. However, adult cat adopters were more likely to have been influenced by price than kitten adopters. Adoption outcomes were generally positive in all age and adoption price groups. This study provides evidence to inform the design of strategies to encourage adult cat adoptions.

**Abstract:**

The percentage of adult cats euthanized in animal shelters is greater than that of kittens because adult cats are less likely to be adopted. This study aimed to provide evidence to inform the design of strategies to encourage adult cat adoptions. One such strategy is to discount adoption prices, but there are concerns that this may result in poor adoption outcomes. We surveyed 382 cat adopters at the time of adoption, to assess potential determinants of adopters’ cat age group choice (adult or kitten) and, for adult cat adopters, the price they are willing to pay. The same respondents were surveyed again 6–12 months after the adoption to compare outcomes between cat age groups and between adult cats in two price categories. Most adopters had benevolent motivations for adopting from the shelter and had put considerable thought into the adoption and requirements for responsible ownership. However, adult cat adopters were more likely to have been influenced by price than kitten adopters. Adoption outcomes were generally positive for both adult cats and kittens and for adult cats adopted at low prices. The latter finding alleviates concerns about the outcomes of “low-cost” adoptions in populations, such as the study population, and lends support for the use of “low-cost” adoptions as an option for attempting to increase adoption rates. In addition, the results provide information that can be used to inform future campaigns aimed at increasing the number of adult cat adoptions, particularly in devising marketing strategies for adult cats.

## 1. Introduction

Every year, many thousands of cats are surrendered to animal shelters globally, including cats surrendered to the RSPCA (Royal Society for the Prevention of Cruelty to Animals), Australia’s largest animal sheltering organisation [1]. For any particular time period, the number of cats reclaimed by their owners or adopted by new owners is less than the number entering shelters; consequently, many cats are euthanized [2,3]. Euthanasia of these animals raises serious ethical issues [4], particularly if they are healthy [5,6], and is of increasing concern to the community [7,8]. In addition, this results in substantial financial costs to the community [3] and is associated with mental health issues for the workers involved [5,6].

The numbers of adult cats and kittens admitted annually to RSPCA shelters in Australia are similar [3,9], but adult cats are less likely to be adopted [2,9,10], and hence, a greater percentage of adult cats are euthanized compared to kittens [2,3,9]. Effective approaches to increase the rate of adoption of adult cats are required, and animal shelters need evidence to inform the design of strategies, such as targeted marketing and promotions, to encourage adult cat adoptions. Knowledge about the people adopting adult cats and kittens and potential determinants of whether people will choose an adult cat or a kitten are an integral part of this evidence base.

One strategy used to increase the numbers of cats adopted is the discounting or waiving of adoption fees [11,12,13]. This strategy has attracted criticism, with concerns that low-cost adoption prices could be associated with devaluation of adopted cats, impulse buying, adoption by unsuitable people and poor outcomes for the cat [11,12,13]. Inherent in these concerns is an assumption that, compared to people adopting cats with high adoption prices, key attributes of people adopting cats at low or no cost differ and that these differences adversely affect the care of the adopted cat and the adoption outcome. These assumptions have not been fully assessed, because although attachment and various outcome measures have been compared between adopters of fee-waived and full-priced cats [11,12,13], no such comparisons have been reported for cats adopted at “low cost”, and adopter characteristics have not been reported for adopters of “low-cost” cats. In addition, if people are to be encouraged to adopt an adult cat rather than a kitten, it is important to have knowledge of the outcomes of both adult cat and kitten adoptions, as this knowledge may help in the design of marketing campaigns for adult cats and kittens.

This study was conducted to provide empirical evidence to inform this debate and guide future campaigns to increase the numbers of cats adopted from shelters. The study aims were to describe selected attributes of adopters of adult cats and kittens, assess potential determinants of whether people choose an adult cat rather than a kitten, assess potential determinants of whether people adopt a low or higher priced adult cat, describe and compare outcomes of adoptions between adult cats and kittens and describe and compare outcomes of adoptions between low-priced and higher-priced adult cats.

## 2. Methods

### 2.1. Study Overview

A cross-sectional study was conducted with a subset of people adopting adult cats and/or kittens from RSPCA Queensland’s animal shelter in Wacol, Australia, between February, 2013, and December, 2013. Adult cats and kittens are referred to collectively as cats, except when referring specifically to a particular cat age group (adult cat or kitten). Data were collected using two questionnaires; one was administered at the time of the adoption and the other 6–12 months after the adoption. Participation was voluntary, and the study was approved by the University of Queensland Ethics Committee (Project Number 2011001160).

At the participating shelter, each cat was classified, based on dentition, as either an adult cat (>4 months of age) or a kitten (≤4 months of age) by a trained staff member. The shelter had implemented a “low-cost” adult cat adoption promotion before the study began and continued this promotion throughout the study period. However, the adoption prices for adult cats varied during the study period. The majority of adult study cats were adopted during a AUD$20 adoption price promotion (199 cats), some during a AUD$99 adoption price promotion (20 cats) and some (13) during an adoption promotion during which adopters could nominate the price they were prepared to pay for the cat (during this promotion, the adoption prices nominated varied from AUD$99 to $250). For reporting and statistical analyses, cats were classified into two adoption price groups: AUD$20 (199 cats) and ≥AUD$99 (33 cats). Adoption price information was unavailable for the remaining 21 adult study cats. All kittens were priced at AUD$180 throughout the study period.

### 2.2. Sample Size

We aimed to recruit 100 cats in each of the three comparison groups (kittens and adult cats adopted for two different prices). This would have resulted in at least 80% statistical power for the detection of differences in mean attachment scores between pairs of comparison groups at the 0.05 level if the within-group standard deviation of mean attachment score was 10.1, as reported by Weiss [11], and the true differences in mean attachment scores between comparison groups were four or more. Assuming attachment scores are normally distributed, a true difference of this magnitude equates to the difference between the 42nd and 58th percentiles. Power calculations were performed using the Compare 2 module (Version 2.69) of WinPepi (Version 11.11; [14]). However, it was not practical to enrol these numbers of cats, so the study power was less than this. After studies are conducted, the effects of sample size are best assessed by examining the precision of effect estimates. This can be assessed using 95% confidence intervals; highly imprecise intervals (as evidenced by wide confidence intervals) are noted.

### 2.3. Participant and Cat Selection and Data Collection

All people over 18 years of age adopting one or more adult cats and/or kittens from the participating shelter during the study period were eligible for enrolment (including those adopting cats that had been previously adopted, returned and made available for adoption again). Those adopting on multiple days during the study period were eligible for enrolment separately on each of those days, but no adopter was enrolled on more than one day. Senior adoption staff at the participating shelter were trained by the researchers to enrol participants using a standardised recruitment methodology, which included providing details about the research aims and study design. These staff selected and trained other adoption staff for participant recruitment. Shelter staff were requested to approach all people adopting one or more cats during the study period, invite them to participate and provide them with an information sheet about the study. Adopters during the study period were also made aware of the study through flyers and notices in the adoption area of the animal shelter. Participants were offered a small toy for their newly adopted cat and the opportunity to win an AUD$100 store voucher. Those who agreed to participate gave written consent and completed a hard copy of the first questionnaire at that time; these responses were subsequently entered into a digitised questionnaire [15].

E-mail addresses and telephone numbers were obtained from participants and were used to contact them six to twelve months after the adoption, at which time they were asked to complete the second questionnaire. Those who provided an e-mail address on their consent form were sent a URL, which linked to the on-line questionnaire. A reminder e-mail was sent two weeks after the initial email if the questionnaire had not been completed. If the questionnaire was not completed within two weeks of the reminder e-mail or the participant provided only a telephone number, the researchers attempted to contact the participant by telephone and have them complete the second questionnaire via telephone interview. Telephone responses were entered directly into a digitised questionnaire (Qualtrics [15]). Those participants who did not respond to the e-mail and had not provided a telephone number were lost to follow-up for the second part of the study. It was assumed for the purposes of the study that the person present at the adoption, who signed the adoption papers and gave consent to be part of the study, was the person who chose the cat and made decisions about the adoption. The shelter’s policy was to not allow the purchase of a cat for another person as a gift or as a proxy, and shelter staff routinely questioned potential adopters at the time of adoption to ensure that this did not occur (personal communication with RSPCA staff [16]).

To calculate response rates, data were obtained from the shelter’s database for all cats adopted during the study period, including the cats’ dates of adoptions, adopters’ names and postcodes and cat age group (*i.e.*, adult cat or kitten). Each person adopting one or more cats on a single day was counted as one adopter for the calculation of the response rate. This was calculated as the number of adopters who were enrolled and completed the first questionnaire during the study period as a proportion of the total number of people who adopted one or more cats from the shelter during the study period. As each record in the shelter’s database represented one cat being adopted, to calculate the total number of people who adopted one or more cats from the shelter during the study period, adopters with the same first name, surname and postcode on the same day were assumed to be the same person and counted as one adopter. Response rates were calculated for all cats pooled and also separately for adult cats and kittens.

### 2.4. Questionnaire Design

The questionnaires were developed based on review of relevant literature and consultation with academic and industry experts. The questionnaires were then tested for validity and reliability by administration to test respondents (not enrolled participants). To assess the questionnaires’ validity, test respondents were able to ask questions and clarify the meaning of questions during their questionnaire session. The questionnaires were then modified based on their feedback. The reliability of the questionnaires were then assessed using a test-retest evaluation; the questionnaires were administered to test respondents at two separate time points, and revisions were made as necessary, based on the repeatability of the test respondents’ responses. The questionnaires contained a combination of forced choice and open-ended questions. The first questionnaire consisted of questions specific to the adopter and the adoption, while the second questionnaire consisted of questions specific to the adopter and questions about each enrolled cat (full details of both questionnaires can be found in Table S1). The first questionnaire consisted of five sections:

1. Respondent demographics and cat ownership history.
2. General attitudes towards cats.
3. Adoption-related considerations: cost-related considerations, including the amount of money the adopter planned to spend on purchasing/adopting a cat before coming to the shelter, other cat sources considered, length of time spent considering the adoption and importance of the lower-than-normal promotional cat adoption price (for adult cat adopters).
4. Factors related to the adoption: each respondent was asked to rate their level of agreement with statements about whether they considered each of a series of possible adoption-related factors when planning to adopt a cat.
5. Reasons for adopting from the animal shelter rather than from another source: each respondent was asked to rate their level of agreement with statements about whether each of a series of possible reasons contributed to their decision to adopt from the participating animal shelter.

For Section 4 and Section 5, respondents were also given the opportunity to provide details about other considerations or reasons in free text fields.

The variables described above were later used as independent variables in the comparisons between cat age groups and adult cat adoption price groups.

The second questionnaire, administered 6–12 months after the adoption, consisted of four sections; each section asked questions about the adopted cat and the cat adopter’s opinion about adopting from the shelter:

1. Cat demographics: Cat sex and hair coat length and whether the cat had a health or behavioural problem.
2. Outcomes of the adoption: cat retention, adopter’s self-rated attachment to the cat, adopter’s satisfaction with the cat, whether the adopter would choose to adopt from the shelter again in the future and the amount of money the adopter would be prepared to pay in the future to adopt another cat from the shelter. In addition to the self-rated measure, the attachment of the respondent to the cat(s) they had adopted was quantified using the Lexington Attachment to Pets Scale (“Attachment Scale”) [17]. Instructions clarified that in the Attachment Scale statements, the term “pet” referred specifically and only to the cat(s) the participant adopted from the RSPCA when they were enrolled in the study. The responses for each of the Attachment Scale statements were allocated scores using the same system as Weiss [11]: strongly disagree (allocated a score of (1)), somewhat disagree (2), somewhat agree (3) and strongly agree (4). These scores were then summed for each cat to give an overall attachment score; with 23 statements in the Attachment Scale, scores could vary from 23 to 92.
3. Caretaking behaviours towards the cat and information about the cat’s lifestyle (for example, indoor/outdoor status).
4. Reasons why some adopters no longer had the adopted cat.

The variables described above were later used as dependent variables and were compared between cat age groups and between adult cat adoption price groups (the independent variables).

Participants who had multiple enrolled cats were asked to complete separate questionnaires for each cat.

### 2.5. Statistical Analyses

All statistical analyses were performed using Stata (Version 12.1, StataCorp, College Station, TX, USA). Distributions of key variables were compared between adopters whose second questionnaire was completed online or via telephone. Since these did not differ substantially, data from the two collection methods were pooled for analyses. Data from the first and second questionnaires for each enrolled cat were matched using unique identification numbers assigned to each adopter/cat combination at the time of data entry of the first questionnaire data. For all analyses of associations, the individual cat was the unit of analysis.

Not all respondents answered all questions. The proportions of adopters and cats are reported as percentages of the number of study adopters or cats, respectively, where the necessary data were available.

#### 2.5.1. Respondent Demographics: Socioeconomic Status

To explore the relationship between socioeconomic status and adoption choice, we used the Index of Relative Socio-economic Advantage and Disadvantage [18]. Each respondent was classified based on the national decile for their home postcode, using indices calculated with the 2011 census data. Thus, the socioeconomic index described the socioeconomic status of the respondent’s home area, rather than that of the respondent’s household. This index was used in the cat age group and adoption price group comparisons as an independent variable.

#### 2.5.2. Differences between the Amount of Money the Adopter Planned to Spend on Purchasing/Adopting a Cat before Coming to the Shelter and Actual Adoption Price Paid

A variable was created to describe the actual price paid for the cat(s) relative to the amount of money the adopter planned to spend on purchasing/adopting a cat before coming to the shelter. Cats were then divided into four groups: cats where the adopter had paid the same, less or more than they had planned and cats for which the adopter had no price in mind. Distributions of cats were reported separately for adult cats and kittens. This variable was used in the cat age group and adoption price group comparisons as an independent variable.

#### 2.5.3. Determinants of Cat Age Group (Adult Cat or Kitten) Adopted

Potential determinants of cat age group adopted (adult cat or kitten) were screened using logistic regression, with adopter fitted as a random effect to account for clustering of cat within adopter; models were fitted using the -xtlogit- command in Stata. All variables from the first questionnaire were screened as independent variables against the dependent variable (cat age group), including respondent demographics, cat ownership history, general attitudes towards cats, adoption-related considerations, factors considered in relation to the adoption and reasons for adopting from the animal shelter (full details of variables screened are provided later). Independent variables collected on the Likert scale were collapsed into three categories for analyses (strongly or somewhat agree, neither agree nor disagree and strongly or somewhat disagree) to avoid, where possible, sparse or zero category combinations. Independent variables with overall *p*-values <0.1 on univariable analysis (eight in total) were then all forced simultaneously into a multivariable model. For each variable that had an overall *p*-value ≥0.1 on univariable analysis, odds ratios were simultaneously adjusted for the eight variables in the multivariable model, by forcing the variable into that model.

#### 2.5.4. Determinants of Adoption Price Paid for Adult Cats

Potential determinants of adoption price paid for adult cats (≥AUD$99 rather than AUD$20) were analysed using the same univariable methods as described above with the adoption price paid for adult cats used as the dependent variable and all other variables from the first questionnaire used as independent variables. The two variables that had overall *p*-values <0.1 on univariable analysis were forced simultaneously into a multivariable model. No significant associations were found using this model, so the results from the univariable models are reported.

#### 2.5.5. Comparisons of Adoption Outcomes between Adult Cats and Kittens

In these comparisons, each variable measuring an adoption outcome was treated as a dependent variable with the distribution of the variable compared using cat age group as the independent variable in univariable analyses.

Distributions of responses for each dependent variable (adoption outcome) (as collected 6–12 months after the cat’s adoption) with more than two ordinal categories were compared between cat age groups using proportional odds models, using the -ologit- command in Stata (full details are provided in the results tables and their footnotes). Robust standard errors that accounted for clustering of cat with adopter were used. The exponentiated coefficients from these models estimated the effects of the adopted cat being an adult cat (rather than a kitten) on the odds of the response being at or above a dependent variable (adoption outcome) category rather than below that category. Proportional odds models are based on the assumption that the ratio of these odds is the same regardless of which dependent variable (adoption outcome) category used as a cut-point (the proportional odds assumption). For each adoption outcome, this assumption was assessed by comparing the log-likelihoods of the proportional odds model and the corresponding multinomial logit model, using the likelihood ratio test without accounting for clustering of cat within adopter. For the respondents’, agreement with “I like cats”, there was evidence of non-proportional odds as indicated by a low *p*-value from the likelihood ratio test (<0.05), so results from the multinomial logistic model (rather than from the proportional odds model) with robust standard errors that accounted for clustering of cat within adopter were used for this variable. Distributions of responses for each binary dependent variable (adoption outcome) (full details are provided in the results tables and their footnotes) were compared between cat age groups using random effects logistic regression, in order to account for clustering of cat within adopter, with the -xtlogit- command in Stata. For one variable (did the adopter intend to keep the cat?), there was a response category-cat age group combination that contained no cats, and so the distribution of this binary variable was compared between cat age groups using exact logistic regression. Conditional probability tests were used; *p*-values were calculated using the mid-P rule as recommended by Agresti [19]. The attachment score was treated as a continuous dependent variable and analysed using linear regression, with adopter fitted as a random effect using Stata’s -xtreg- command.

#### 2.5.6. Comparisons of Adoption Outcomes between Adult Cats Adopted for Different Prices

Distributions of responses for each adoption outcome for adult cats were compared between adoption price categories (≥AUD$99 or AUD$20) using the same univariable approaches as described immediately above. In these comparisons, adoption outcomes for adult cats were treated as dependent variables with their distributions compared using adoption price categories as the independent variable. For four variables (the respondent’s type of accommodation, did the adopter intend to keep the cat, the frequency of the adopter holding/stroking/cuddling the cat/kitten and did the adopter put external identification on the cat), there was a response category-adoption price category combination that contained no cats, and so, the distributions of these variables were compared between adoption price categories using exact logistic regression as described above.

## 3. Results

In total, 1804 people adopted cats from the participating shelter during the study period (1,001 adult cats and 809 kittens); of these, 382 adopters (21%) were enrolled in the study and completed the first questionnaire at the time they adopted. Of the 998 people who adopted adult cats from the participating shelter during the study period, 248 were enrolled (25%). Of the 811 kitten adopters, 134 were enrolled (17%) (five people adopted an adult cat and a kitten). The majority of respondents adopted just one cat (*n* = 375), but seven adopted two cats each (five adopted two adult cats and two adopted two kittens); all 389 cats were enrolled (248 adult cats, 134 kittens and seven cats whose type was not recorded).

Of the 644 adult cats that were adopted from the shelter during the study period for $20, 199 were enrolled (31%), and of the 259 adult cats that were adopted for between AUD$99–250, 33 were enrolled (13%). The remaining adult cats adopted from the shelter during the study period were adopted for <AUD$20 (26), from AUD$20–98 (67) or more than AUD$250 (5).

The second questionnaire, administered 6–12 months after the adoption, was completed by 70% (266/382) of the enrolled adopters; 164 out of 248 adult cat adopters (66%), 97 out of 134 kitten adopters (73%) and five out of seven adopters for which the cat age group adopted was unknown (71%). The second questionnaire was completed online by 210 respondents and through a telephone interview by 56. Reasons that were given for not completing the second questionnaire were that no or incorrect contact details (six respondents), the adopter no longer wanted to participate or was unable to participate (five respondents) and the adopter could not be contacted within the study time frame (105 respondents). Of the 389 cats enrolled at adoption, second questionnaires were completed for 271 cats; 68% of adult cats (168/248), 73% of kittens (98/134) and 71% of cats for which the cat age group was unknown (5/7). Those cats for which the cat age group information was unavailable were excluded from the reporting of the adoption outcomes by age group, leaving responses available for 168 adult cats and 98 kittens. Of the responses for adult cats, 157 had adoption price information. Those cats for which the adoption price information was unavailable were excluded from the reporting of the adoption outcomes by adoption price group, leaving responses available for 138 adult cats in the AUD$20 group and 19 in the ≥AUD$99 group.

### 3.1. Respondent Demographics, Cat Ownership History and General Attitudes towards Cats

The majority of study adopters were female (73%; 272/373), were aged between 25 to 45 years (59%; 220/373) and were employed full- or part-time (61%; 227/373). The over-representation of females among the study adopters was consistent with participant demographics reported in other research in this field [20,21]. The median socioeconomic advantage disadvantage index decile was eight for both the study adopters and the entire population of adopters from the shelter during the study period; a higher index value indicates that the postcode is relatively advantaged (Table 1, Table S2 and Table S3).

Almost half of the respondents (48% or 172/362) had previously owned cats that they obtained from only a non-welfare source (for example, a pet shop or breeder, as described in Table S1); 27% of respondents (99/362) had previously owned cats that they obtained from only a welfare source (for example, an animal shelter); 10% of respondents (38/362) had previously owned cats that they obtained from both a welfare and non-welfare source; and 15% of respondents (53/362) had not owned a cat before. The majority of respondents somewhat or strongly agreed that they liked cats (91%; 345/379).

**Table 1:** Distributions of cats by cat age group (adult cat or kitten) and associations between potential determinants of cat age group adopted for 389 cats adopted from an animal shelter in Australia in 2013 ^1^.

Independent Variable and Categories	Adult Cats *n* (%) ^2^	Kittens *n* (%) ^2^	Adjusted Odds Ratio ^3^	95% Confidence Interval	Adjusted *p*-Value ^4^
**Index of relative socioeconomic advantage disadvantage decile (*n* = 376)**					**0.06**
8–10	155 (63)	89 (69)	Reference category		
4–7	48 (20)	32 (25)	0.0	0.0–3.9	0.17
1–3	43 (18)	9 (7)	4.4	0.1–194.2	0.44
**Agreement with the statement “I like cats” (*n* = 379)**					**<0.01**
Somewhat or strongly agree	233 (94)	112 (85)	Reference category		
Did not agree	14 (6)	20 (15)	0.0	0.0–0.8	0.04
**Source of previously owned cats (*n* = 362)**					**0.24**
Non-welfare source (e.g., pet shop, breeder)	114 (49)	58 (45)	Reference category		
Welfare source (e.g., animal shelter, municipal pound/council animal control centre)	66 (28)	33 (26)	1.7	0.1–38.2	0.75
Never owned a cat before	25 (11)	28 (22)	0.0	0.0–4.2	0.17
Both welfare and non-welfare source	29 (12)	9 (7)	1.7	0.0–78.5	0.78
**Amount of money the adopter planned to spend on purchasing/adopting a cat before coming to the shelter (*n* = 378)**					**<0.01**
≤$50	45 (18)	19 (14)	Reference category		
$51–150	61 (25)	20 (15)	17.0	0.1–2,405.4	0.27
≥$151	68 (28)	58 (44)	0.0	0.0–1.2	0.06
No price in mind	73 (30)	34 (26)	0.2	0.0–10.8	0.43
**Agreement with the statement “When I was considering purchasing/adopting a cat leading up to today, I considered the following factors…”**					
*“The initial purchase price of a cat/kitten” (n = 365)*					**0.21**
Strongly or somewhat agree	106 (44)	64 (52)	Reference category		
Neither agree nor disagree	69 (29)	41 (33)	1.6	0.1–32.8	0.75
Somewhat or strongly disagree	66 (27)	19 (15)	24.8	0.3–2,479.6	0.17
*“My preferred cat/kitten breed (e.g., purebred or crossbreed)” (n = 355)*					**0.05**
Somewhat or strongly agree	52 (22)	43 (35)	Reference category		
Neither agree nor disagree	92 (40)	45 (37)	26.9	0.3–2,644.6	0.16
Strongly or somewhat disagree	89 (38)	34 (28)	39.3	0.3–5,313.5	0.14
**Agreement with the statement “I chose to adopt this cat/kitten from an animal shelter rather than a breeder, pet shop or other source because…”**					
*“My friends or family thought I should get a cat/kitten from an animal shelter” (n =328)*					**<0.01**
Somewhat or strongly agree	62 (30)	50 (43)	Reference category		
Neither agree nor disagree	94 (45)	52 (44)	120.1	0.7–19,532.2	0.07
Strongly or somewhat disagree	55 (26)	15 (13)	412.4	1.4–118,576.7	0.04
*“The cat/kitten was cheaper from the shelter than from other sources” (n = 310)*					**<0.01**
Somewhat or strongly agree	78 (38)	29 (27)	Reference category		
Neither agree nor disagree	82 (40)	45 (42)	0.0	0.0–1.3	0.06
Strongly or somewhat disagree	43 (21)	33 (31)	0.0	0.0–0.3	0.02

^1^ All variables that had an overall *p*-value <0.1 on univariable analysis were simultaneously forced into a multivariable model. The results presented here are from that multivariable model; all eight independent variables fitted in that model are reported here. Results for variables that had an overall *p*-value ≥0.1 on univariable analysis are reported in Table S2. ^2^ Total numbers of respondents differ between exposure variables, as not all respondents answered each question, and within variables, percentages do not always sum to 100% due to rounding. ^3^ The odds ratio estimates the odds of an adopter adopting an adult cat rather than a kitten. Odds ratios are adjusted for all other variables in the model (*i.e.*, for all other exposure variables reported in this table). Two hundred fifty nine cats were included in the multivariable model, as those with missing values for any of these exposure variables were excluded. ^4^ Bolded values are overall likelihood ratio test *p*-values for the variable; non-bolded values are Wald *p*-values for the specific category, relative to the reference category. *p*-Values are adjusted for all other variables in the model.

### 3.2. Adoption-Related Considerations

The majority of cats’ adopters had been thinking about adopting a cat for over one month (82%; 308/374). The price of the cat was an important consideration for some, but for most, price was of less importance than finding the right animal (56%; 211/375) or price was not a consideration when selecting a cat (33%; 123/375) (Table S2).

Many cats’ adopters (40%; 148/375) had considered a source other than the shelter to get a cat. Of these sources, the most commonly considered were other welfare options, such as another animal shelter/welfare animal welfare organization (*n* = 65), municipal pound/council animal control centre (*n* = 16) or private cat rescue/rehoming group (*n* = 53), but some cats’ adopters had also considered non-welfare sources, such as a pet shop (*n* = 35), family/friends (*n* = 9), breeder (*n* = 31) or advertisements in the local paper or on the Internet (*n* = 35) (respondents could indicate one or more sources that they considered).

### 3.3. Factors Related to the Adoption

**Figure 1:**
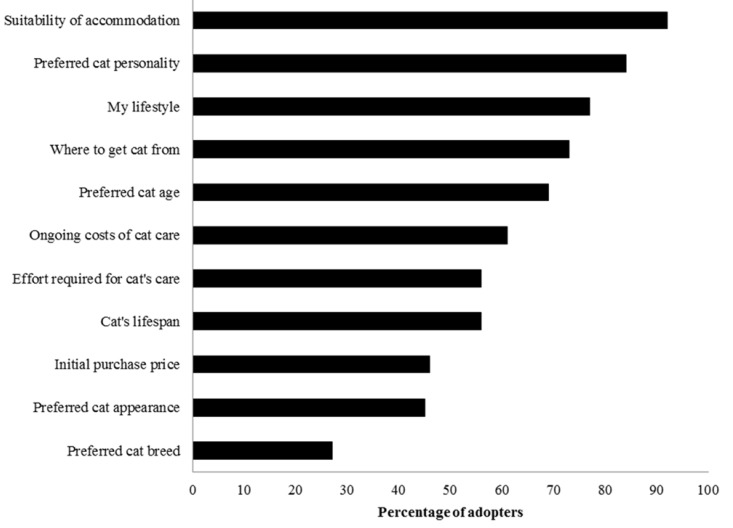
Factors considered by adopters before adopting their study cat(s). Between 355 and 367 of the 382 study adopters answered each question. Each adopter was asked to rate whether they had considered each of these factors before adopting their study cat(s) from the animal shelter; those who answered somewhat or strongly agree were classified as having considered that factor before adopting.

Most cats’ adopters had considered a range of factors related to the adoption (Table 1, Figure 1 and Table S2 and Table S3); the three most common were the suitability of their accommodation for a cat, preferred cat personality and their lifestyle.

### 3.4. Reasons for Adopting from the Animal Shelter Rather than from Another Source

**Figure 2:**
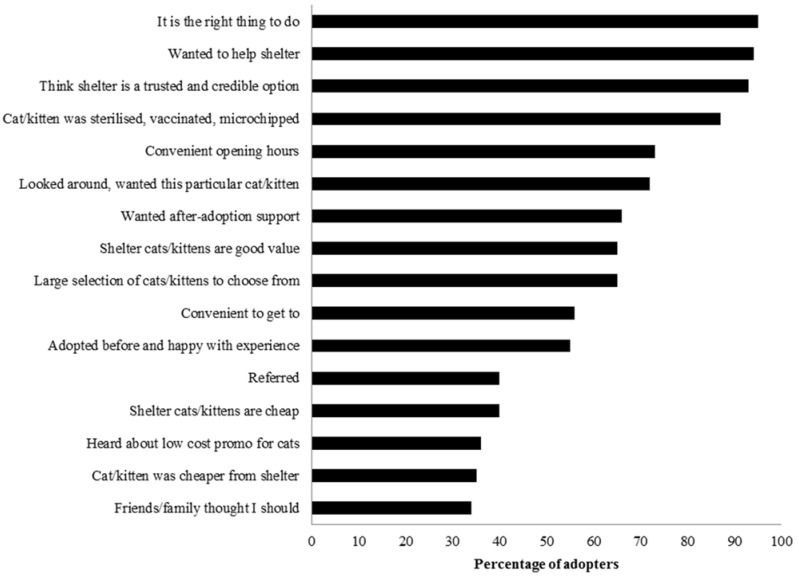
Reasons that contributed to adopters’ decision to adopt their study cat(s) from the animal shelter rather than from another source. Between 267 and 370 of the 382 study adopters answered each question. Each adopter was asked to rate whether each of these reasons contributed to their decision to adopt their study cat(s) from the animal shelter rather than from another source; those who answered somewhat or strongly agree were classified as having had that reason contribute to their decision.

Most cats’ adopters also had a range of reasons for adopting from the shelter rather than getting their cat from another source (Table 1, Figure 2 and Table S2 and Table S3); the three most common were that the adopter felt that adopting from a shelter was the right thing to do, they thought that the shelter was a trusted and credible option and they wanted to help the shelter.

### 3.5. Differences between the Amount of Money the Adopter Planned to Spend on Purchasing/Adopting a Cat before Coming to the Shelter and Actual Adoption Price Paid

Differences between the amount of money the adopter planned to spend on purchasing/adopting a cat before coming to the shelter (“planned spend”) and actual adoption price paid were known for 364 of the 389 study cats (94%). The adopter paid approximately the same as what they were planning to spend for 44% of kittens (58/131) and 24% of adult cats (55/233); the adopter paid less for no kittens and for 43% of adult cats (101/233); and the adopter paid more for 30% of kittens (39/131) and 3% of adult cats (6/233). The adopter had no price in mind initially for 26% of kittens (34/131) and 31% of adult cats (71/233).

### 3.6. Determinants of Cat Age Group (Adult Cat or Kitten) Adopted

Associations between variables that were included in the multivariable model of cat age group adopted (those with overall *p*-value of <0.1 on initial univariable screening analysis) are reported in Table 1. Results for all other variables that had an overall *p*-value ≥0.1 on initial univariable screening analysis are reported in Table S2, with odds ratios and *p*-values adjusted for all eight variables included in the multivariable model reported in Table 1.

Multivariable modelling revealed that respondents who adopted from the shelter because cats are less expensive there, and those who indicated a greater liking for cats were more likely to be an adopter of an adult cat rather than a kitten (Table 1). Respondents who adopted from the shelter because friends/family thought they should were more likely to be an adopter of a kitten rather than an adult cat. Planned spending was also correlated with cat age group adopted, with those who had a higher planned spending or no price in mind more likely to also be an adopter of a kitten than an adult cat.

### 3.7. Determinants of Adoption Price Paid for Adult Cats

There were no significant differences detected in any exposure variables between adopters of adult cats in different adoption price groups, but the effect estimates were imprecise (as evidenced by wide confidence intervals) (Table S3). Forty-nine percent (67/137) of adult cat adopters agreed that hearing about the lower than normal cat adoption price promotion was a reason for them adopting from the shelter (Table S3). Twelve percent of adult cat adopters indicated that they considered the lower than normal promotional cat adoption price as very or extremely important in their decision to adopt a cat; 36% considered it somewhat important (50/137); and 51% (70/137) did not consider the lower than normal promotional cat adoption price as important.

### 3.8. Cat Demographics

Of the cats for which cat demographic questions were completed, 51% were female (136/266), 63% were short haired (168/268), 31% medium haired (83/268) and 6% long haired (17/269). Fifty cats (19%; 50/266) had a health problem, and 14 cats (5%; 14/267) had a behavioural problem for which, at the time of the adoption, the cat’s adopter signed a waiver form for the problem (to confirm that they had been made aware of it and the implications and were prepared to proceed with the adoption anyway).

### 3.9. Adoption Outcomes

Attachment scores were approximately normally distributed with a mean of 67 (range 40–92, SD 10), indicating strong attachment for most cats [11]. For the majority of cats (88%; 225/257), their adopter also self-rated themselves as very attached to the cat. Six to 12 months after adoption, for almost all cats, the adopter intended to keep the cat (99%; 226/229) and agreed that they would choose to adopt from the shelter again in the future if they wanted to adopt another cat (95%; 234/246; Table S4 and Table S5).

For most cats, the adopter undertook most caretaking behaviours (application of flea/tick medication, de-worming, registration, putting a collar on the cat and confinement of the cat), and for 89% (229/258) of cats, the adopter intended to take the cat to the veterinarian yearly (Table S4 and Table S5). Most cats (87%; 225/259) were allowed inside their adopter’s house all of the time and were held/stroked/cuddled daily (98%; 253/258; Table S4 and Table S5).

Nineteen cats (7%; 19/266) were no longer in the care of the adopter when they completed the second questionnaire. These cats had various outcomes, including being returned to the shelter (2%; 5/266), dying (2%; 4/266), running away (1%; 2/266), relationship breakdown and the other person had the cat (1%; 2/266) and the cat was given or sold to another person (2%; 7/266). The percentage of adopted cats that were returned to the shelter within a month of the adoption (the shelter’s return rate) over the same period of time was 5% (96/1,809), not including the study cats. Of the adult cats adopted at low-price (AUD$20; 199 cats) and higher-price (≥AUD$99; 33 cats), one and zero respectively had been returned to the shelter.

### 3.10. Comparisons of Adoption Outcomes between Adult Cats and Kittens

Comparisons of adoption outcomes between adult cats and kittens are reported in Table 2 (results with significant differences) and Table S4 (results with non-significant differences). Although most adopters were satisfied with the cat (96%; 247/258), a significant association was found between satisfaction with the cat and cat age group, with adopters of adult cats more likely than adopters of kittens to be satisfied rather than very satisfied (Table 2). Kitten adopters were more likely to be prepared to pay a higher adoption price for a cat in the future compared to adopters of adult cats (Table 2). There were no other significant differences detected in any variables between adopters of adult cats and kittens in univariable analyses, but the effect estimates were imprecise (as evidenced by wide confidence intervals) (Table S4).

There were no significant differences (*p* ≥ 0.05) detected in attachment score, self-rated attachment, adopter’s intention to keep the cat, adopter’s willingness to adopt from the shelter in the future between adult cats and kittens in univariable analyses, but again, effect estimates were imprecise (Table S4).

There were no significant (*p* < 0.05) differences detected in caretaking behaviours shown towards the cat between cat age groups in univariable analyses, but these effect estimates were also imprecise.

**Table 2:** Distributions of adoption outcomes by age group of cat adopted (adult cat or kitten) for 271 cats adopted from an animal shelter in Australia in 2013 ^1^.

Dependent Variable (Adoption Outcome) and Categories	Adult Cats *n* (%) ^2^	Kittens *n* (%) ^2^	Odds Ratio ^3^	95% Confidence Interval ^4^	*p*-Value ^5^
**Satisfaction with the Adopted cat (*n* = 258)**			2.2 ^4^	1.0–4.7	**0.04**
Very satisfied	131 (78)	81 (89)			
Satisfied	28 (17)	7 (8)			
Neither satisfied or dissatisfied	3 (2)	1 (1)			
Dissatisfied ^5^	5 (3)	2 (2)			
**Amount of money the adopter planned to spend on purchasing/adopting a cat from the shelter in the future (*n* = 246)**			2.3	1.4–3.8	**<0.01**
≥$201	27 (17)	24 (28)			
$101–200	62 (39)	44 (51)			
$21–100	69 (43)	17 (20)			
≤$20	1 (1)	2 (2)			

^1^ Variables with an overall *p*-value <0.05 on univariable analysis; results for variables that had an overall *p*-value ≥0.05 on univariable analysis are reported in Table S4. ^2^ Total numbers of respondents differ between variables, as not all respondents answered each question, and within variables, percentages do not always sum to 100% due to rounding. ^3^ Odds ratio estimate; this estimates the odds of an adopter choosing any particular category for adult cats relative to those for kittens. ^4^ Bolded values are overall likelihood ratio test *p*-values for a variable; non-bolded values are Wald *p*-values. ^5^ Includes “dissatisfied” and “very dissatisfied”.

### 3.11. Comparisons of Adoption Outcomes between Adult Cats Adopted for Different Adoption Prices

Comparisons of adoption outcomes between adult cats adopted at different adoption prices are reported in Table S5. No significant differences (*p* ≥ 0.05) were detected in attachment score, self-rated attachment, adopter’s intention to keep the cat, adopter’s willingness to adopt from the shelter in the future and in caretaking behaviours shown towards the cat between adult cats adopted for different adoption prices in univariable analyses, but again, effect estimates were imprecise (Table S5).

## 4. Discussion

Adoption outcomes were generally positive for both adult cats and kittens and for adult cats adopted at low prices from this shelter. The finding that outcomes for cats adopted at low prices are generally good alleviates concerns about the outcomes of “low-cost” adoptions in populations such as the study population. This lends support for the use of “low-cost” adoptions as an option for attempting to increase adoption rates. However, adverse effects of cat age group or cat price on adoption outcomes cannot be excluded, as the 95% confidence intervals were wide and included odds ratios that could be indicative of adverse outcomes for some variables. Adoption outcomes in our study were measured by attachment of the adopter to the cat, whether the adopter would adopt another cat from the shelter in the future, the amount of money the adopter would be prepared to pay for a cat from the shelter in the future, satisfaction with the adoption and caretaking/lifestyle of the cat. Measures of adoption success other than the traditional shelter-based measurement of return rates were used in this study, as return rates seemed likely to be only a limited measure of adoption success. Indeed, even if the cat is not returned to the shelter, an adoption may still not be successful (*i.e.*, if the adopter keeps the cat, but is not satisfied with the adoption, not attached to the cat, does not provide a good level of care or gives the cat away to another person, shelter, municipal pound or rescue group). Our results support this approach, as only five of the fifteen cats no longer in the care of the adopter when they completed the second questionnaire (and not having died when under care of the adopter) were returned to the shelter, while six cats had been given or sold to another person.

The majority of the adopters in our study, including those who adopted cats for the “low-cost” adoption fee of AUD$20, had high attachment scores, indicative of a strong attachment to their cat. A lack of association between financial resources and attachment to pets [17,22,23] is supported in other studies by the similar attachment scores found regardless of price paid for the cat [11,12,13]. A decision to introduce “low-cost” adoption promotions at an animal shelter should be based on two main considerations: (1) whether the outcomes of the low-cost adoptions are satisfactory; and (2) whether adoption promotions result in an overall increase in cats adopted. The results of this study have positive implications for the use of “low-cost” adoption campaigns, as they address the first consideration and show that adoption outcomes are generally positive for cats adopted during a “low-cost” adoption price promotion. The results from this study also showed that almost half of adult cat adopters indicated that hearing about the lower than normal cat adoption price promotion was a reason for them adopting from the shelter and considered the lower than normal promotional cat adoption price as important in their decision to adopt a cat. It could be inferred from these findings that almost half the adopters were attracted by the low adoption price and may not have adopted otherwise. This suggests that low-cost adoption promotions may result in some increase in adoptions, but further work is needed to fully address the second consideration mentioned above.

The majority of adopters in our study seemed to have put substantial thought into the adoption process, irrespective of whether they then chose to adopt an adult cat or kitten or the adoption price paid. Adopter’s decisions regarding the adoption had less to do with price and more to do with finding a cat that was suitable for them. The majority of adopters had been thinking of adopting a cat for a substantial period of time and had considered a wide variety of responsible ownership factors prior to the adoption (such as their lifestyle, suitability of their accommodation for a cat and ongoing costs of cat care). These findings alleviate potential concerns that low-cost adoption promotions may attract unsuitable adopters or result in impulse buying without due consideration [11,12,13].

Benevolent motivations for adopting from the shelter were evident in the studied population, with over 75% of adopters choosing to adopt from the shelter because they felt it was the right thing to do, thought that the shelter was a trusted and credible option or wanted to help the shelter. In addition, those adopters who had considered another source mostly considered other welfare organisations. These findings provide insights that can help inform the design of novel strategies to encourage shelter adoptions. Examples include actively promoting the idea that adopting from the shelter is an altruistic action that adopters will feel good about and developing non-monetary reward systems to both reward adopters and motivate them to share information about their adoption experience. Rewards, including non-monetary rewards, are recognised as a way to encourage information sharing in organisations [24,25,26]. Based on the findings in this study, a similar system could work well for animal welfare organisations to encourage adoptions in general and to specifically encourage the adoption of particular animal groups that are often overlooked, such as adult cats (for example, by offering greater “rewards” for adopting or recommending adult cats).

Some significant differences were found between adult cat and kitten adopters in their reasons for adopting from the shelter; this information may also assist the design of strategies to encourage the adoption of adult cats. Compared to kitten adopters, adult cat adopters were more likely to have planned to spend less money on the adoption, suggesting that price sensitivity may be associated with the choice to adopt an adult cat. Shelters could utilise this knowledge to promote adult cat adoptions through advertising that focuses not only on the low adoption price of adult cats, but also on the other price-related benefits of adopting an adult cat (for example, that routine veterinary visits/vaccinations should only be required annually compared to a kitten, which will need a series of routine veterinarian visits to be fully vaccinated). A better understanding of the importance of price to adopters and its interaction with other factors in the adoption decision making process would be helpful for shelters to determine how best to price their cats and kittens and achieve a sustainable revenue without introducing negative impacts on either population. Such an understanding would also improve the accuracy of modelling designed to assist shelter managers maintain the financial health of the organisation, while implementing strategies, such as low-cost adoptions, which are expected to increase the number of adoptions [27]. A number of findings from this study suggest that price sensitive adopters are more likely to adopt adult cats rather than kittens; many adopters of adult cats considered the lower than normal promotional adoption price important, and people who chose to adopt from the shelter because cats were less expensive there were more likely to be adopters of adult cats than kittens. Additionally, planned spending was correlated with cat age group adopted, with those who had higher planned spending or no price in mind more likely to also be an adopter of a kitten than an adult cat. However, because we used a cross-sectional design, we could not measure the adopters’ planned spending before adoption, and it is possible that some adopters’ planned spending may have been altered by the actual adoption price they paid. Additionally, it is not possible to know the direction of causality of the relationship between planned spending and cat choice (*i.e.*, does planned spending influence cat choice/price, or does cat choice/price influence planned spending, or is the relationship more complex?). Further work would be necessary to explore this issue. Recording potential adopters’ planned spending prior to the decision to adopt a specific cat may help to elucidate the relationship.

People with a more positive attitude towards cats tended to adopt adult cats rather than kittens. A possible explanation for this finding is that positive experiences from previous cat ownership translated into a greater willingness to adopt an adult cat. In keeping with the finding that benevolent motivations predominated amongst our adopter population, adopters with positive attitudes towards cats may have been motivated by concern that the adults were less likely to be adopted [2,10]. Perhaps people who were less sure of whether they liked cats were more likely to adopt a kitten, because they thought that would be the best introduction to cat ownership.

Some significant differences in outcomes after adoption were found for adult cats compared to kittens. Almost all adopters were satisfied with their adopted cat regardless of whether it was an adult or kitten, but adopters of adult cats were more likely to be just satisfied (rather than very satisfied) than adopters of kittens. This difference is unlikely to be of concern for animal welfare organisations attempting to find homes for cats, since an adopter being satisfied with the cat is nevertheless a positive outcome. However, further research may elucidate the underlying reasons for this and may yield useful information for animal welfare organisations regarding potential issues with integrating an adult cat into a home and how to prevent or manage any issues.

Kitten adopters were prepared to pay a higher adoption price for a cat in the future, but almost all kitten adopters paid a higher adoption price for their kitten compared to adult cat adopters. Consequently, this finding may indicate that future spending is influenced more by actual price paid than by cat age group. This is consistent with marketing theory on pricing decisions being constructed, in part, from an internal reference price based on previous experiences [28]. For example, if an adopter pays $20 to adopt a pet and is satisfied with the pet, in the future, that person might be likely to assume that it is not necessary to pay more than $20 to adopt a satisfactory pet. Conversely, if an adopter pays $180 to adopt a pet and is satisfied with the pet, in the future, that adopter might be likely to assume that one needed to pay $180 or more to adopt a satisfactory pet. This may have implications for animal welfare organisations’ future decision making about how to price adoption fees, as adopters who have paid a “low” adoption price may expect to always pay a similar “low” price in future. This may limit the ability of the shelter to vary prices over the long term, especially for returning “customers”.

We found no significant associations between adoption price paid and cat caretaking behaviours, echoing the results of other studies in the USA [12,13,29]. However, further research is necessary to assess whether there is an association between adoption price paid and cat caretaking behaviours, as effect estimates and associated confidence intervals were not reported in the USA studies, and the effect estimates in our study were very imprecise. The quality of care provided to a pet has been found to be more influenced by owner characteristics—the owner’s gender, level of education, previous cat and ownership experiences—than attachment and price paid for the pet [11,30,31] and is reportedly more reliant on the owner’s willingness to spend money on the pet rather than on the income of the owner [23]. The kind of person adopting from a shelter may differ from those who obtain a cat from other sources [11], and this difference may be amplified by the screening process used by shelters to determine if a person is a suitable adopter. It is possible that our adopter population may have been more homogeneous in terms of demographics, income, previous cat ownership or cat lifestyle than the general population and that these factors do differ between adopters of different cat age groups and adopters of cats with different adoption prices in more diverse adopter populations.

We generated overall attachment scores for each cat by allocating numerical scores of one to four to the ordinal responses to each of the 23 statements in the Lexington Attachment to Pets Scale [11,17]. These scores were then summated for each cat to give an overall attachment score, and we treated these overall scores as continuous data, as in previous research [11]. This approach is valid only if each incremental increase in score for each statement has the same underlying meaning. If this is not the case, the same overall attachment scores for different cats may have different inherent meaning, cats with the same degree of attachment may have different scores and statistical methods treating these scores as continuous data, as we have done, are invalid. There is a need to validate this approach to assessing attachment with “gold standard” continuous measures of adoption.

### Limitations

The study’s initial intention was to compare adult cats adopted at “normal” adoption prices with those adopted at “low” adoption prices and to assess the success of the adoption promotion at increasing adoption rates by comparing these before, during and after the adoption promotion. However, for reasons outside of our control, the adoption price promotions were introduced at the participating shelter before the study began and were continued throughout the study period. Therefore, it was not possible to collect data that would have allowed precise comparisons between “normal” and “low” adoption price groups or to compare adoption rates before, during and after the adoption promotion. In addition, the shelter changed location, premises and operating procedures in the months before the study, making a comparison of adoption rates with the same period in the preceding year invalid. However, although adult cat adoption prices were always promotional and never “normal” during the study period, the adoption price for cats did vary during different promotions (there was a AUD$20 adoption price promotion, a AUD$99 adoption price promotion and an adoption promotion during which the adoption prices varied from AUD$99 to 250). The data obtained nonetheless allowed us to assess adoption outcomes for cats adopted at “low” adoption prices. It is not possible to determine from this study if outcomes might be better for adult cats adopted at higher prices, but considering that outcomes for “low-cost” cats were generally good, better results for higher priced cats would need to be very good indeed. Nevertheless, any potential difference between good and very good outcomes is unlikely to be of great concern for animal welfare organisations attempting to find homes for cats, as it is likely that a good outcome for an adopted cat is all that is sought by the organisation, and outcomes were generally good for cats adopted at “low cost” in this study.

An increased adoption rate during a fee-waived adoption promotion has been reported [11], but more work is needed to demonstrate that “low-cost” adoption promotions would also increase adoption rates as has been proposed [27]. If both “low-cost” adoption promotions and fee-waived adoption promotions are equally successful at increasing adoption rates, shelters may want to charge a small fee for the adoption to help the shelter recoup some of the costs incurred.

The timeframe for completion of the second questionnaire varied from 6–12 months post adoption. Some follow-up interviews were completed at six months post adoption to facilitate the completion of the study within the study period. The time frame available for the study meant that the longest follow-up time possible was a maximum of 12 months from the time of adoption. It would be useful to follow-up adopted cats over longer time periods to determine whether the positive outcomes demonstrated in the short term are still evident in the long term. It would also have been ideal to standardize post adoption responses to a shorter window of time, but this was not possible due to the limited time frame available for the study, the difficulty in contacting some of the participants and the limited time available to conduct interviews and follow-up with participants.

Numbers of adult cats *versus* kittens present in the shelter during the study period may have influenced some of our results; if there were fewer kittens available, this may create a perception of increased value and, hence, an increased likelihood of “purchase”, even though the “purchase price” was higher (similar to consumer reactions to perceived limited availability of merchandise [32,33,34]). In order to assess this potential confounder, in future research, the ratio of number of adults/kittens available for adoption each day to the number of adults/kittens adopted on each day could be taken into consideration when analysing other variables.

The financial environment could potentially affect motivations for adoption (for example, the importance of price-related motivations may differ depending on available discretionary spending money) and the kind of people adopting cats during a specific period (for example, some people may only have discretionary spending money in certain financial circumstances, such as after tax return or pension/welfare payouts), and these factors may, in turn, affect the outcome for the cat. It is not possible to control for this, but the prolonged study period, which covered almost an entire year, should have mitigated any such effect. Both the study sample and the general population of adopters at the shelter had an above-average socioeconomic index score, which may have influenced the results. Therefore, inferences from the results of this study might only apply to other such areas where the socioeconomic status is higher than average.

It is possible that some study adopters answered questions dishonestly because they perceived that there was a “right” answer; social normative pressure may make them want to appear more benevolent or a “better” adopter/owner than they actually are (social desirability bias [35]). However, self-administered questionnaires, as used in this study, may decrease this kind of false reporting, as people perceive more anonymity [21].

The aim was to recruit 100 cats in each of the three comparison groups (kittens and adult cats adopted for two different prices), but the final numbers that were enrolled were lower than this. If it had been possible to enrol 100 cats in each of the three comparison groups, the study power and precision of effect estimates (odds ratios) would have been greater than that achieved, if all else were equal. The low response rate in this study, although comparable to other similar studies [11,21,36], created a potential for selection bias, as more committed and caring adopters may have been more likely to participate. This kind of bias is unavoidable, as voluntary participation is an ethical necessity [21]. The offer of a cat toy and prize was aimed at engaging a broader range of participants and helping minimise this bias. One problem in attempting to recruit sufficient participants in this study was that every adopter was not approached and asked to participate in the study, as intended. Many of the people assisting with adoptions were volunteers and were not briefed about the study and, so, did not approach any adopters to invite them to participate. In addition, during busy periods, adoption staff forgot to ask adopters to participate. For future studies of this nature, it would be ideal to have a dedicated research team member present to ask adopters if they would participate throughout the study period. This would likely result in an improved participation rate. The return rate for study cats was lower than the shelter’s return rate for the same period, suggesting some degree of selection bias with those participants who had retained their study cats possibly being more likely to have answered the second questionnaire. People who had returned their adopted cat to the shelter may have been reluctant to answer the second questionnaire, because they were distressed or felt guilty about returning the cat or because they felt that they may have been judged for returning the cat.

## 5. Conclusions

The majority of cat adoption outcomes in the shelter population studied were positive for both adult cats and kittens and for adult cats adopted at low prices. These findings should allay concerns that “low-cost” cat adoptions will have poor outcomes and, in addition, demonstrate that both adult cat and kitten adoptions are generally successful. Most adopters from this shelter had benevolent motivations for adopting and put considerable thought into the adoption and responsible ownership. This study provides information that can be used to guide strategies aimed at increasing adoptions, particularly of adult cats. Our findings should encourage shelters to be creative with adoption and other marketing campaigns and to consider options, such as “low-cost” cat adoption promotions if ongoing use of these promotions is shown to increase the numbers of cats adopted.

## Supplementary Materials

**Table S1:** Questionnaire categories and data variable details.

Categories	Variable Details
**First questionnaire (administered at the time of the adoption)**	
Demographics	Respondent gender, age, occupation status (e.g., employed full-time, unemployed *etc.*), postcode (postcode was later used to determine the index of relative socio-economic advantage or disadvantage for the respondent (Australian Bureau of Statistics, 2011)), relationship status (e.g., whether the respondent was married, single or in a *de facto* relationship where a couple is not legally married but have a relationship as a couple living together on a genuine domestic basis), child status (e.g., whether the respondent had children living with them 50% or more of the time), household income (e.g., double or single income or pension), housing situation (e.g., renting or homeowner), type of accommodation (e.g., house or apartment), number of people living in the household (e.g., children or other adults). All these were categorical questions with the response category details for each given in Tables S2 and Table S3.
Previous cat ownership history	Source of previously owned cats (respondents were given a list of response options to choose from, e.g., RSPCA, another shelter, a breeder *etc*. and other and were able to give further details in a free text field. For reporting and analysis these options were later simplified into four categories: (1) a non-welfare source (breeder, pet shop, friend, relative acquaintance, advertisement, obtained unintentionally as abandoned at their property or a stray, or children brought the cat home, private sale, left with them by another family member); (2) a welfare source (animal shelter, municipal pound/council animal control centre, private cat rescue/re-homing group); (3) never owned a cat before; and (4) both a non-welfare and welfare source (for those people who had owned multiple cats and had obtained them from sources that fitted both categories).
General attitude toward cats	The respondent’s level of agreement with the statement “I like cats” (measured on a Likert scale of 1–5 where 1 = strongly disagree and 5 = strongly agree). This was later collapsed into two dependent variable (adoption outcome) categories (agree or did not agree) as the responses were dichotomous and highly polarised.
**Adoption-related considerations**	
Cost-related considerations	- “Planned spending”: the amount of money the adopter planned to spend on purchasing/adopting a cat before coming to the shelter (respondents were given price range options to choose from; see Tables S2 and Table S3 for details of the categories). - The importance of price as a consideration in the adoption (respondents were given options to choose from, see Tables S2 and Table S3 for details of the categories).
Other cat sources considered	- Options were: breeder; pet shop; friend, family member, acquaintance or neighbour; animal shelter other than the RSPCA; municipal pound/council animal control centre; private cat rescue/re-homing group; through an advertisement, e.g., in the local paper or on the Internet; other (respondent was asked to give more details). - For analyses the other cat sources considered were simplified into two categories: did not consider a source other than the shelter or did consider a source other than the shelter.
Length of time spent considering the adoption	Options were: spur of the moment, <1 month (but not spur of the moment), ≥1–<6 months, ≥6–<12 months and ≥12 months.
Importance of the lower than normal promotional adult cat adoption price	Adopters of adult cats were asked how important the lower than normal promotional cat adoption price was in their decision to purchase/adopt the cat (measured on a Likert scale of 1–3 where 1 = not important, 2 = somewhat important and 3 = very or extremely important).
Factors considered related to the adoption	Each respondent was also asked to rate whether they considered each of a series of possible adoption-related factors when planning to adopt a cat/kitten (measured on a Likert scale of 1–5 where 1 = strongly disagree and 5 = strongly agree, these options were later collapsed to three categories: disagree, neither agree nor disagree and agree): suitability of their accommodation for a cat/kitten; initial purchase price of a cat/kitten;, ongoing costs to care for a cat/kitten; preferred cat/kitten breed (e.g., purebred or crossbreed); preferred age of cat (e.g., kitten or adult); preferred cat/kitten appearance (e.g., colour, coat length); preferred cat/kitten personality (e.g., playful, placid, independent, affectionate); level of effort involved in caring for the cat/kitten (e.g., grooming, daily maintenance); a cat/kitten's lifespan and therefore the duration of care required for the cat/kitten; their lifestyle; and where to get the cat/kitten from.
Reasons for adopting from the animal shelter	Each respondent was asked to rate whether each of a series of possible reasons contributed to their decision to adopt from the shelter kitten (measured on a Likert scale of 1–5 where 1 = strongly disagree and 5 = strongly agree, these options were later collapsed to three categories: disagree, neither agree nor disagree and agree): “cats/kittens from shelters are good value”; “cats/kittens from shelters are cheap compared to cats/kittens from other sources”; “I think adopting cats/kittens from shelters is the right thing to do”; “shelter cats/kittens are already sterilised, vaccinated, microchipped, checked by a vet and treated for parasites”; “friends or family thought I should get a cat/kitten from an animal shelter”; “there is large selection of cats/kittens to choose from at an animal shelter”; “I have adopted a cat/kitten from an animal shelter previously and was happy with the experience”; “the shelter's opening hours are convenient for me”; “the shelter is convenient for me to get to”; “the shelter is a trusted and credible option”; “by getting a cat/kitten from the shelter I help the shelter”; “I was referred to the shelter by a friend, relative, colleague or acquaintance”; “I wanted the support given after purchase/adoption by the shelter”; ‘I had heard about the promotion the shelter was having for sale/adoption of cats”; “the cat/kitten was cheaper at the shelter than other sources”; and “I looked around and liked this particular cat/kitten at the shelter”.
**Second questionnaire (administered 6–12 months after adoption)**	
Cat demographics	Cat sex and hair coat length and whether the cat had a health or behavioural problem for which a waiver form was signed at the time of adoption. The details of the response categories are shown in Tables S2 and Table S3.
Cat retention	Was the cat was still with the adopter? (response options were: yes or no)
Outcome of the adoption	- The adopter’s self-rated attachment to the cat (measured on a Likert scale of 1–3 where 1 = very attached and 3 = not at all attached). - The adopter’s satisfaction with the cat (measured on a Likert scale of 1–5 where 1= very dissatisfied and 5 = very satisfied). - Would the adopter choose to adopt from the shelter again in the future if they wanted another cat? (response options were: yes, no or unsure) - The amount of money the adopter would be prepared to pay in the future to adopt another cat from the shelter (respondents were given price range options to choose from, the details of the response categories are shown in Tables S4 and Table S5). - Lexington Attachment to Pets Scale questions (details explained in methods).- Did the adopter intend to keep the cat? (response options were: yes, no or unsure)
Cat caretaking and lifestyle	- The frequency of the adopter’s interactions with the cat (holding/stroking/cuddling) (respondents were given options to choose from, the details of the response categories are shown in Tables S4 and Table S5). - Frequency that the cat was allowed inside the house (respondents were given options to choose from, the details of the response categories are shown in Tables S4 and Table S5). - Indoor/outdoor status of the cat (respondents were given options to choose from, the details of the response categories are shown in Tables S4 and Table S5). - Had the adopter put a collar and external identification on the cat, checked the microchip registration details? (Respondents were given options to choose from, the details of the response categories are shown in Tables S4 and Table S5). - Did the adopter intend to take the cat to the vet yearly? (As the second questionnaire was administered 6–12 months after the adoption it was not possible to ask if the cat had been taken for yearly vet visits/vaccinations as the yearly check would not have been due yet. Therefore adopters were asked if they intended to take their cat to the vet for yearly visits and consequently this is only an approximation of intent rather than actual actions) (response options were: yes, no or don’t know). - Frequency of flea/tick medication application and de-worming the cat. (Respondents were given options to choose from, the details of the response categories are shown in Tables S4 and Table S5).
Factors related to the adopter no longer having their adopted cat	- Only asked of those adopters who no longer had their cat. - The adopters who no longer had their cat were asked why they no longer had the adopted cat (options given were “I returned the cat to the shelter”, “The cat passed away”, “I surrendered the cat to another shelter, rescue group or municipal pound/council animal control centre”, “I gave or sold the cat to another person”, “The cat ran away”, “I adopted the cat with another person but we no longer live together and the other person now has the cat” and an option to write in a free text field any other reason.

**Table S2:** Distributions of cats by cat age group (adult cat or kitten) and associations between potential determinants of cat age group adopted for 389 cats adopted from an animal shelter in Australia in 2013 (for those variables which had a *p*-value ≥0.1 on initial univariable screening analysis).

Independent Variable and Categories	Adult Cats *n* (%) ^2^	Kittens *n* (%) ^2^	Adjusted Odds Ratio ^3^	95% Confidence Interval	Adjusted *p*-Value ^4^
**Respondent gender (*n* = 380)**					**0.83**
Male	68 (27)	34 (36)	Reference category		
Female	180 (73)	98 (74)	0.7	0.0–13.1	0.83
**Respondent age (*n* = 378)**					**0.90**
18–25	52 (21)	24 (18)	Reference category		
26–35	81 (34)	38 (29)	1.3	0.0–50.3	0.88
36–45	63 (26)	41 (31)	1.0	0.0–41.4	0.98
46–55	28 (11)	18 (14)	0.4	0.0–50.8	0.74
56–65	15 (6)	9 (7)	0.1	0.0–20.2	0.35
≥66	7 (3)	2 (2)	5.1	0.0–31,196.7	0.71
**Respondent occupation status (*n* = 380)**					**0.97**
Employed full-time	177 (47)	66 (50)	Reference category		
Employed part-time	33 (13)	17 (13)	1.8	0.0–143.4	0.79
Casual worker	18 (7)	5 (4)	24.5	0.0–14,447.4	0.33
Homemaker	25 (10)	16 (12)	0.8	0.0–47.3	0.92
Student	21 (8)	11 (8)	0.4	0.0–32.9	0.67
Retired	10 (4)	5 (4)	1.2	0.0–815.4	0.96
Self-employed	17 (7)	6 (5)	31.4	0.0–29,387.3	0.32
Unemployed	5 (2)	4 (3)	1.0	0.0–432.1	1.00
Other	2 (1)	2 (2)	61.2	0.0–1.5e+07	0.51
**Respondent relationship status (*n* = 354)**					**0.14**
Married	102 (44)	47 (39)	Reference category		
Single	72 (31)	39 (32)	0.4	0.0–12.7	0.56
*De facto* relationship	49 (21)	29 (24)	0.0	0.0–1.1	0.05
Widowed or divorced	9 (4)	7 (6)	0.0	0.0–4.4	0.14
**Respondent’s household income (*n* = 378)**					**0.60**
Double income	126 (51)	63 (48)	Reference category		
Single income	107 (44)	63 (48)	0.3	0.0–4.0	0.33
Pension	13 (5)	6 (5)	1.4	0.0–267.5	0.89
**Children under 15 years of age living with the respondent (*n* = 378)**					**1.00**
None	126 (51)	63 (48)	Reference category		
One or more	120 (49)	69 (52)	0.9	0.1–11.3	0.94
**Respondent’s housing situation (*n* = 378)**					**0.34**
Homeowner	144 (59)	81 (61)	Reference category		
Renting	85 (35)	45 (34)	0.2	0.0–5.4	0.35
Other	17 (7)	6 (5)	6.8	0.0–1,589.5	0.49
**Respondent’s type of accommodation (*n* = 380)**					**0.20**
House	191 (77)	109 (86)	Reference category		
Apartment/unit/townhouse/studio	45 (18)	20 (15)	1.0	0.0–33.1	0.98
Farm/hobby farm/other	9 (3)	3 (2)	2,023.9	0.1–6.87e+7	0.15
**Number of people in the respondent’s household (*n* = 360)**					**1.00**
1	30 (13)	11 (9)	Reference category		
2–3	116 (50)	64 (51)	0.3	0.0–3.2	0.29
≥4	88 (38)	51 (41)	0.2	0.0–3.0	0.25
**The importance of price as a consideration in the adoption (*n* = 375)**					**0.19**
Price of the cat was part of the picture, but it was more about finding the right animal	138 (57)	73 (55)	Reference category		
Price was not a consideration when I selected a cat	77 (32)	46 (35)	0.1	0.0–2.7	0.16
I had a set budget to purchase the cat that I could not go over	18 (7)	10 (8)	15.8	0.0–12,193.1	0.42
I wanted the best value/cheapest option to purchase a cat	10 (4)	3 (2)	6.8	0.0–9,177.0	0.60
**Other cat sources considered (*n* = 361)**					**0.50**
Did not consider a source other than the shelter	141 (60)	72 (57)	Reference category		
Did consider a source other than the shelter	93 (40)	55 (43)	0.3	0.0–7.9	0.49
**Length of time spent considering the adoption (*n* = 374)**					**0.75**
Spur of the moment	9 (4)	5 (4)	Reference category		
< 1 month	34 (14)	18 (14)	138.3	0.1–378,474.3	0.22
≥1–<6 months	93 (38)	52 (39)	127.5	0.1–126,033.0	0.18
≥6–<12months	55 (23)	30 (23)	238.6	0.1–537,146.7	0.16
≥12 months	51 (21)	27 (21)	143.0	0.1–236,717.6	0.19
**Agreement with the statement “When I was considering purchasing/adopting a cat leading up to today, I considered the following factors…”**					
**“Suitability of my accommodation for a cat/kitten” (*n* = 367)**					**0.70**
Somewhat or strongly agree	220 (91)	118 (94)	Reference category		
Neither agree nor disagree	12 (5)	3 (2)	23.7	0.0–7,712,852.0	0.63
Strongly or somewhat disagree	10 (4)	4 (3)	3.1	0.0–935,508.8	0.86
**“Ongoing costs to care for a cat/kitten” (*n* = 366)**					**0.35**
Somewhat or strongly agree	141 (59)	85 (68)	Reference category		
Neither agree nor disagree	60 (25)	22 (18)	12.4	0.2–642.9	0.21
Strongly or somewhat disagree	39 (16)	19 (15)	2.9	0.0–293.7	0.66
**“My preferred age of cat (e.g., kitten or adult)” (*n* = 364)**					**1.00**
Somewhat or strongly agree	159 (67)	93 (74)	Reference category		
Neither agree nor disagree	49 (21)	25 (20)	1.7	0.3–10.7	0.55
Strongly or somewhat disagree	30 (13)	8 (6)	12.7	0.5–332/2	0.13
**“My preferred cat/kitten appearance (e.g., colour, coat length)” (*n* = 361)**					**1.00**
Somewhat or strongly agree	97 (41)	65 (52)	Reference category		
Neither agree nor disagree	85 (36)	47 (38)	1.3	0.1–23.1	0.86
Strongly or somewhat disagree	55 (23)	12 (10)	18.0	0.2–1,663.3	0.21
**“My preferred cat/kitten personality (e.g., playful, placid, independent, affectionate)” (*n* = 361)**					**1.00**
Somewhat or strongly agree	193 (81)	108 (87)	Reference category		
Neither agree nor disagree	29 (12)	11 (9)	0.6	0.0–23.7	0.78
Strongly or somewhat disagree	15 (6)	5 (4)	4.4	0.0–1,339.4	0.61
**“The level of effort involved in caring for the cat/kitten (e.g., daily maintenance)” (*n* = 364)**					**1.00**
Somewhat or strongly agree	127 (53)	79 (64)	Reference category		
Neither agree nor disagree	74 (31)	30 (24)	1.9	0.1–35.8	0.68
Strongly or somewhat disagree	40 (17)	14 (11)	3.4	0.1–128.0	0.51
**“The cat/kitten’s lifespan and therefore the duration of care required” (*n* = 365)**					**1.00**
Somewhat or strongly agree	132 (55)	70 (57)	Reference category		
Neither agree nor disagree	64 (27)	34 (27)	0.8	0.1–12.6	0.9
Strongly or somewhat disagree	45(19)	20 (16)	0.8	0.0–20.1	0.9
**“My lifestyle” (*n* = 361)**					**1.00**
Somewhat or strongly agree	186 (79)	93 (75)	Reference category		
Neither agree nor disagree	36 (15)	24 (19)	0.2	0.0–3.8	0.27
Strongly or somewhat disagree	15 (6)	7 (6)	0.1	0.0–11.2	0.38
**“Where to get the cat/kitten from” (*n* = 362)**					**1.00**
Somewhat or strongly agree	169 (71)	96 (77)	Reference category		
Neither agree nor disagree	49 (21)	22 (18)	2.0	0.1–52.5	0.69
Strongly or somewhat disagree	20 (8)	6 (5)	1.5	0.0–111.8	0.87
**Agreement with the statement “I chose to adopt this cat/kitten from an animal shelter rather than a breeder, pet shop or other source because…”**					
**“Cats/kittens from shelters are good value” (*n* = 362)**					**1.00**
Somewhat or strongly agree	153 (65)	83 (66)	Reference category		
Neither agree nor disagree	53 (23)	31 (25)	0.9	0.0–20.7	0.96
Strongly or somewhat disagree	30 (13)	12 (10)	8.2	0.1–664.2	0.35
**“Cats/kittens from shelters are cheap” (*n* = 355)**					**0.89**
Somewhat or strongly agree	95 (41)	47 (38)	Reference category		
Neither agree nor disagree	92 (40)	54 (44)	0.5	0.0–11.2	0.65
Strongly or somewhat disagree	44 (19)	23 (19)	0.4	0.0–48.5	0.72
**“I think adopting cats/kittens from shelters is the right thing to do” (*n* = 363)**					**0.36**
Somewhat or strongly agree	223 (94)	122 (97)	Reference category		
Neither agree nor disagree	5 (2)	2 (2)	96.2^5^	0.0–2,616,438.0	0.38
Strongly or somewhat disagree	9 (4)	2 (2)			
**“Shelter cats/kittens are already sterilised, vaccinated, microchipped, checked by a vet and treated for parasites” (*n* = 364)**					**0.77**
Somewhat or strongly agree	206 (87)	112 (88)	Reference category		
Neither agree nor disagree	20 (8)	12 (10)	0.2	0.0–12.2	0.44
Strongly or somewhat disagree	11 (5)	3 (2)	0.4	0.0–271.9	0.77
**“There is large selection of cats/kittens to choose from at an animal shelter” (*n* = 357)**					**0.29**
Somewhat or strongly agree	152 (66)	78 (62)	Reference category		
Neither agree nor disagree	59 (26)	40 (32)	0.1	0.0–2.2	0.14
Strongly or somewhat disagree	20 (9)	8 (6)	0.1	0.0–14.2	0.31
**“I have adopted a cat/kitten from an animal shelter previously and was happy with the experience” (*n* = 267)**					**0.57**
Somewhat or strongly agree	96 (56)	50 (52)	Reference category		
Neither agree nor disagree	38 (22)	29 (30)	0.1	0.0–8.7	0.33
Strongly or somewhat disagree	37 (22)	17 (18)	0.3	0.0–56.2	0.67
**“The shelter’s opening hours are convenient for me” (*n* = 364)**					**0.84**
Somewhat or strongly agree	172 (73)	95 (75)	Reference category		
Neither agree nor disagree	47 (20)	27 (21)	0.4	0.0–12.2	0.60
Strongly or somewhat disagree	18 (8)	5 (4)	1.9	0.0–748.0	0.84
**“The shelter is convenient for me to get to” (*n* = 369)**					**0.28**
Somewhat or strongly agree	139 (58)	69 (58)	Reference category		
Neither agree nor disagree	52 (22)	32 (25)	0.1	0.0–2.1	0.13
Strongly or somewhat disagree	50 (21)	27 (21)	0.5	0.0–17.0	0.70
**“The shelter is a trusted and credible option” (*n* = 369)**					**0.96**
Somewhat or strongly agree	223 (93)	122 (95)	Reference category		
Neither agree nor disagree	10 (4)	3 (2)	0.4	0.0–429.5	0.79
Strongly or somewhat disagree	8 (3)	3 (2)	3.2	0.0–106,515.9	0.83
**“By getting a cat/kitten from the shelter I help the shelter” (*n* = 370)**					**0.85**
Somewhat or strongly agree	229 (95)	120 (94)	Reference category		
Neither agree nor disagree	6 (3)	5 (4)	0.1	0.0–70.5	0.54
Strongly or somewhat disagree	7 (3)	3 (2)	1.7	0.0–13,456.0	0.91
**“I was referred to the shelter by a friend, relative, colleague or acquaintance” (*n* = 309)**					**0.76**
Somewhat or strongly agree	42 (21)	15 (14)	Reference category		
Neither agree nor disagree	81 (40)	45 (42)	0.4	0.0–38.6	0.70
Strongly or somewhat disagree	78 (39)	48 (44)	0.2	0.0–18.2	0.50
**“I wanted the support given after purchase/adoption by the shelter” (*n* = 352)**					**0.04**^6^
Somewhat or strongly agree	157 (69)	75 (61)	Reference category		
Neither agree nor disagree	53 (23)	42 (34)	0.0	0.0–0.9	0.04
Strongly or somewhat disagree	19 (8)	6 (5)	4.6	0.0–681.3	0.55
**“I had heard about the promotion the shelter was having for sale/adoption of cats” (*n* = 277)**					**0.29**
Somewhat or strongly agree	74 (39)	26 (30)	Reference category		
Neither agree nor disagree	63 (33)	35 (40)	0.1	0.0–3.3	0.18
Strongly or somewhat disagree	53 (28)	26 (30)	0.2	0.0–11.8	0.45
**“I looked around and liked this particular cat/kitten at the shelter” (*n* = 326)**					**0.83**
Somewhat or strongly agree	156 (72)	80 (74)	Reference category		
Neither agree nor disagree	33 (15)	14 (13)	0.5	0.0–19.9	0.74
Strongly or somewhat disagree	29 (13)	14 (13)	0.3	0.0–12.5	0.50

^1^ All variables that had an overall *p*-value ≥0.1 on univariable analysis are presented in this table. In order to adjust the odds ratios for the variables that were significant, each of the variables in this table were adjusted for the eight variables in the multivariable model reported in Table 1. Two hundred forty six cats were included in the model, as those with missing values for any of the eight independent variables in the multivariable model were excluded. ^2^ Total numbers of respondents differ between exposure variables, as not all respondents answered each question, and within variables, percentages do not always sum to 100% due to rounding. ^3^ The odds ratio estimates the odds of an adopter adopting an adult cat rather than a kitten. ^4^ Bolded values are the overall likelihood ratio test *p*-values for the variable; non-bolded values are Wald *p*-values for the specific category, relative to the reference category. ^5^ Categories combined for analysis as sparse or zero cells did not allow analysis of the categories separately; indicated by a common vertical line showing the categories that were pooled for analysis. ^6^ In the univariable analysis, *p* = 0.12.

**Table S3:** Distributions of adult cats by adoption price and associations between potential determinants of cat adoption price for 248 adult cats adopted from an animal shelter in Australia in 2013 ^1^.

Independent Variable and Categories	≥AUD$99 *n* (%) ^2^	AUD$20 *n* (%) ^2^	Odds Ratio ^3^	95% Confidence Interval	*p*-Value ^4^
**Respondent gender (*n* = 232)**					**0.38**
Male	8 (24)	56 (28)	Reference category		
Female	25 (76)	143 (72)	4.9	0.2–165.2	0.38
**Respondent age (*n* = 230)**					**0.23**
18–25	4 (13)	45 (23)	Reference category		
26–35	9 (28)	66 (33)	2.3	0.2–26.4	0.50
36–45	7 (22)	55 (28)	2.1	0.2–26.1	0.57
46–55	6 (19)	20 (10)	13.2	0.7–267.8	0.09
56–65	4 (13)	10 (5)	28.9	0.7–1,121.5	0.07
≥66	2 (6)	2 (1)	245.1	0.7–90,590.6	0.07
**Respondent occupation status (*n* = 323)**					**0.12**
Employed full time	17 (52)	92 (46)	Reference category ^5^		
Employed part time	5 (15)	28 (14)			
Casual worker	0	17 (9)			
Self-employed	2 (6)	15 (8)			
Homemaker	3 (9)	20 (10)	0.9	0.1–11.6	0.91
Retired	5 (15)	2 (1)	138.3^5^	2.2–8,559.6	0.02
Other	0	2 (1)			
Student	0	19 (10)	0.1^5^	0.0–8.3	0.28
Unemployed	1 (3)	4 (2)			
**Index of relative socioeconomic advantage disadvantage decile (*n* = 230)**					**0.45**
8–10	24 (73)	120 (61)	Reference category		
4–7	5 (15)	38 (19)	0.4	0.0–4.0	0.43
1–3	4 (12)	39 (20)	0.2	0.0–3.0	0.27
**Respondent relationship status (*n* = 220)**					**0.76**
Married	14 (42)	87 (47)	Reference category^5^		
*De facto* relationship	7 (21)	40 (21)			
Single	10 (30)	53 (28)	1.4	0.1–17.9	0.81
Widowed or divorced	2 (6)	7 (4)	5.8	0.1–640.3	0.46
**Respondent’s household income (*n* = 230)**					**0.18**
Double income	14 (42)	105 (53)	Reference category		
Single income	15 (46)	85 (43)	1.7	0.3–8.5	0.51
Pension	4 (12)	7 (4)	26.3	0.8–870.0	0.07
**Children under 15 years of age living with the respondent (*n* = 230)**					**0.08**
None	22 (67)	94 (48)	Reference category		
One or more	11 (33)	103 (52)	0.2	0.0–1.2	0.08
**Respondent’s housing situation (*n* = 231)**					**0.12**
Homeowner	25 (76)	109 (55)	Reference category		
Renting	7 (21)	74 (37)	0.1	0.0–1.0	0.05
Other	1 (3)	15 (8)	0.1	0.0–11.4	0.29
**Respondent’s type of accommodation (*n* = 232) ^6^**					**0.86**
House	25 (76)	152 (76)	Reference category		
Apartment/unit/townhouse/studio	7 (21)	36 (18)	1.2	0.4–2.9	0.72
Farm/hobby farm/other	1 (3)	11 (6)	0.6	0.0–3.5	0.84
**Number of people in the respondent’s household (*n* = 219)**					**0.14**
1	6 (20)	22 (12)	Reference category		
2–3	19 (63)	89 (47)	0.6	0.0–10.9	0.70
≥4	5 (17)	78 (41)	0.0	0.0–1.6	0.09
**Source of previously owned cats (*n* = 219)**					**0.93**
Non-welfare source	14 (45)	97 (52)	Reference category		
Welfare source	9 (29)	50 (27)	1.6	0.2–11.8	0.64
Never owned a cat before	4 (13)	20 (11)	2.0	0.1–29.9	0.62
Both welfare and non-welfare source	4 (13)	21 (11)	1.9	0.1–27.0	0.64
**Amount of money the adopter planned to spend on purchasing/adopting a cat before coming to the shelter (*n* = 231)**					**0.17**
≤$50	3 (9)	40 (20)	Reference category		
$51–150	10 (30)	45 (23)	3.2	0.7–14.0	0.12
≥$151	12 (40)	50 (25)	3.8	0.9–16.0	0.07
No price in mind	7 (21)	63 (32)	1.5	0.3–6.8	0.58
**The importance of price as a consideration in the adoption (*n* = 228)**					**0.90**
Price of the cat was part of the picture, but it was more about finding the right animal	18 (60)	113 (57)	Reference category		
Price was not a consideration when I selected a cat	9 (30)	60 (30)	0.9	0.1–5.9	0.93
I had a set budget to purchase the cat that I could not go over	0	18 (7)	0.5^5^	0.0–8.4	0.65
I wanted the best value/cheapest option to purchase a cat	3 (10)	7 (4)			
**Other cat sources considered (*n* = 218)**					**0.39**
Did not consider a source other than the shelter	20 (67)	108 (58)	Reference category		
Did consider a source other than the shelter	10 (33)	80 (43)	0.4	0.1–3.1	0.39
**Length of time spent considering the adoption (*n* = 227)**					**0.60**
Spur of the moment	0	8 (4)	Reference category^5^		
< 1 month	3 (10)	28 (14)			
≥1–<6 months	14 (45)	71 (36)	5.3	0.3–82.9	0.24
≥6–<12months	6 (20)	47 (24)	2.1	0.1–39.1	0.61
≥12 months	8 (26)	42 (21)	5.1	0.3–97.5	0.28
**Importance of the lower than normal promotional cat adoption price (*n* = 137)**					**0.09**
Not important	20 (77)	50 (45)	Reference category		
Somewhat important	5 (19)	45 (41)	0.1	0.0–0.8	0.04
Very or extremely important	1 (4)	16 (14)	0.0	0.0–4.4	0.15
**Agreement with the statement “I like cats” (*n* = 231)**					**0.40**
Somewhat or strongly agree	31 (94)	190 (96)	Reference category		
Did not agree	2 (6)	8 (4)	9.2	0.1–1,551.2	0.40
**Agreement with the statement “When I was considering purchasing/adopting a cat leading up to today, I considered the following factors…”**					
**“Suitability of my accommodation for a cat/kitten” (*n* = 227)**					**0.73**
Somewhat or strongly agree	27 (90)	181 (92)	Reference category		
Neither agree nor disagree	1 (3)	9 (5)	0.5	0.0–44.5	0.77
Strongly or somewhat disagree	2 (7)	7 (4)	4.4	0.1–238.3	0.47
**“The initial purchase price of a cat/kitten” (*n* = 226)**					**0.30**
Somewhat or strongly agree	9 (30)	92 (47)	Reference category		
Neither agree nor disagree	11 (37)	53 (27)	4.2	0.6–31.3	0.16
Strongly or somewhat disagree	10 (33)	51 (26)	3.8	0.5–28.3	0.20
**“Ongoing costs to care for a cat/kitten” (*n* = 225)**					**0.30**
Somewhat or strongly agree	13 (46)	123 (62)	Reference category		
Neither agree nor disagree	9 (32)	45 (23)	3.6	0.5–25.7	0.20
Strongly or somewhat disagree	6 (21)	29 (15)	4.1	0.4–40.5	0.22
**“My preferred cat/kitten breed (e.g., purebred or crossbreed)” (*n* = 219)**					**0.77**
Somewhat or strongly agree	8 (28)	43 (23)	Reference category		
Neither agree nor disagree	10 (35)	78 (41)	0.5	0.1–4.1	0.48
Strongly or somewhat disagree	11 (38)	69 (36)	0.7	0.1–6.3	0.75
**“My preferred age of cat (e.g., kitten or adult)” (*n* = 224)**					**0.48**
Somewhat or strongly agree	22 (79)	129 (66)	Reference category		
Neither agree nor disagree	3 (11)	44 (23)	0.2	0.0–3.3	0.23
Strongly or somewhat disagree	3 (11)	23 (12)	0.6	0.0–11.2	0.72
**“My preferred cat/kitten appearance (e.g., colour, coat length)” (*n* = 224)**					**0.68**
Somewhat or strongly agree	10 (35)	82 (42)	Reference category		
Neither agree nor disagree	11 (38)	70 (36)	1.7	0.3–10.3	0.58
Strongly or somewhat disagree	8 (28)	43 (22)	2.4	0.3–18.6	0.39
**“My preferred cat/kitten personality (e.g., playful, placid, independent, affectionate)” (*n* = 223)**					**0.62**
Somewhat or strongly agree	26 (87)	154 (80)	Reference category		
Neither agree nor disagree	2 (7)	27 914)	0.2	0.0–4.6	0.32
Strongly or somewhat disagree	2 (7)	12 (6)	1.0	0.0–21.9	0.97
**“The level of effort involved in caring for the cat/kitten (e.g., grooming, daily maintenance)” (*n* = 226)**					**0.22**
Somewhat or strongly agree	17 (55)	105 (54)	Reference category		
Neither agree nor disagree	6 (19)	62 (32)	0.3	0.0–2.9	0.32
Strongly or somewhat disagree	8 (26)	28 (14)	3.7	0.4–37.1	0.26
**“The cat/kitten’s lifespan and therefore the duration of care required” (*n* = 226)**					**0.68**
Somewhat or strongly agree	16 (55)	110 (56)	Reference category		
Neither agree nor disagree	6 (21)	51 (26)	0.7	0.1–4.6	0.70
Strongly or somewhat disagree	7 (24)	36 (18)	1.9	0.3–13.4	0.53
**“My lifestyle” (*n* = 222)**					**0.65**
Somewhat or strongly agree	25 (86)	151 (78)	Reference category		
Neither agree nor disagree	3 (10)	31 (16)	0.4	0.0–4.7	0.44
Strongly or somewhat disagree	1 (4)	11 (6)	0.3	0.0–20.8	0.57
**“Where to get the cat/kitten from” (*n* = 224)**					**0.32**
Somewhat or strongly agree	26 (87)	135 (70)	Reference category		
Neither agree nor disagree	2 (7)	42 (22)	0.0	0.0–3.1	0.15
Strongly or somewhat disagree	2 (7)	17 (9)	0.4	0.0–9.1	0.53
**Agreement with the statement “I chose to adopt this cat/kitten from an animal shelter rather than a breeder, pet shop or other source because…”**					
**“Cats/kittens from shelters are good value” (*n* = 221)**					**0.56**
Somewhat or strongly agree	17 (59)	125 (65)	Reference category		
Neither agree nor disagree	9 (31)	42 (22)	2.7	0.4–19.3	0.31
Strongly or somewhat disagree	3 (10)	25 (13)	0.8	0.1–12.0	0.86
**“Cats/kittens from shelters are cheap” (*n* = 218)**					**0.23**
Somewhat or strongly agree	7 (24)	81 (43)	Reference category		
Neither agree nor disagree	15 (52)	73 (39)	5.9	0.7–48.4	0.10
Strongly or somewhat disagree	7 (24)	35 (19)	5.8	0.5–67.8	0.16
**“I think adopting cats/kittens from shelters is the right thing to do” (*n* = 223)**					**0.37**
Somewhat or strongly agree	27 (90)	182 (94)	Reference category		
Neither agree nor disagree	2 (7)	3 (2)	26.1	0.3–2,538.6	0.16
Strongly or somewhat disagree	1 (3)	8 (4)	0.7	0.0–40.5	0.86
**“Shelter cats/kittens are already sterilised, vaccinated, microchipped, checked by a vet and treated for parasites” (*n* = 222)**					**0.63**
Somewhat or strongly agree	27 (90)	166 (87)	Reference category		
Neither agree nor disagree	1 (3)	17 (9)	0.1	0.0–11.4	0.40
Strongly or somewhat disagree	2 (7)	9 (5)	1.9	0.1–56.5	0.72
**“My friends or family thought I should get a cat/kitten from an animal shelter” (*n* = 190)**					**0.59**
Somewhat or strongly agree	10 (42)	63 (38)	Reference category		
Neither agree nor disagree	11 (46)	66 (40)	1.1	0.2–6.6	0.95
Strongly or somewhat disagree	3 (13)	37 (22)	0.3	0.0–4.2	0.35
**“There is large selection of cats/kittens to choose from at an animal shelter” (*n* = 217)**					**0.84**
Somewhat or strongly agree	18 (67)	124 (65)	Reference category		
Neither agree nor disagree	6 (22)	50 (26)	0.7	0.1–4.6	0.73
Strongly or somewhat disagree	3 (11)	16 (8)	1.7	0.1–23.9	0.68
**“I have adopted a cat/kitten from an animal shelter previously and was happy with the experience” (*n* = 160)**					**0.80**
Somewhat or strongly agree	11 (58)	77 (55)	Reference category		
Neither agree nor disagree	3 (16)	32 (23)	0.5	0.0–6.4	0.57
Strongly or somewhat disagree	5 (26)	32 (23)	1.3	0.1–12.1	0.83
**“The shelter’s opening hours are convenient for me” (*n* = 222)**					**0.62**
Somewhat or strongly agree	19 (66)	142 (74)	Reference category		
Neither agree nor disagree	7 (24)	38 (20)	1.9	0.3–13.7	0.53
Strongly or somewhat disagree	3 (10)	13 (7)	3.6	0.2–71.5	0.41
**“The shelter is convenient for me to get to” (*n* = 226)**					**0.43**
Somewhat or strongly agree	16 (55)	115 (58)	Reference category		
Neither agree nor disagree	9 (31)	40 (20)	2.5	0.4–15.7	0.32
Strongly or somewhat disagree	4 (14)	42 (21)	0.5	0.1–4.5	0.54
**“The shelter is a trusted and credible option” (*n* = 226)**					**0.53**
Somewhat or strongly agree	27 (90)	183 (93)	Reference category		
Neither agree nor disagree	0	8 (4)	2.6^5^	0.1–52.5	0.53
Strongly or somewhat disagree	3 (10)	5 (3)			
**“By getting a cat/kitten from the shelter I help the shelter” (*n* = 227)**					**0.79**
Somewhat or strongly agree	27 (93)	187 (94)	Reference category		
Neither agree nor disagree	0	6 (3)	1.6^5^	0.1–38.9	0.79
Strongly or somewhat disagree	2 (7)	5 (3)			
**“I was referred to the shelter by a friend, relative, colleague or acquaintance” (*n* = 309)**					**0.59**
Somewhat or strongly agree	3 (13)	37 (22)	Reference category		
Neither agree nor disagree	11 (46)	66 (40)	4.1	0.3–66.6	0.32
Strongly or somewhat disagree	10 (42)	63 (38)	3.9	0.2–64.7	0.35
**“I wanted the support given after purchase/adoption by the shelter” (*n* = 214)**					**0.66**
Somewhat or strongly agree	21 (75)	123 (66)	Reference category		
Neither agree nor disagree	5 (18)	47 (25)	0.4	0.1–3.2	0.38
Strongly or somewhat disagree	2 (7)	16 (9)	0.6	0.0–13.3	0.72
**“I had heard about the promotion the shelter was having for sale/adoption of cats” (*n* = 180)**					**0.92**
Somewhat or strongly agree	8 (36)	62 (39)	Reference category		
Neither agree nor disagree	8 (36)	50 (32)	1.5	0.2–11.0	0.72
Strongly or somewhat disagree	6 (27)	46 (29)	1.0	0.1–8.6	0.99
**“The cat/kitten was cheaper from the shelter than from other sources” (*n* = 192)**					**0.24**
Somewhat or strongly agree	7 (28)	67 (40)	Reference category		
Neither agree nor disagree	14 (56)	63 (37)	5.4	0.6–45.8	0.12
Strongly or somewhat disagree	4 (16)	37 (22)	1.1	0.1–16.3	0.95
**“I looked around and liked this particular cat/kitten at the shelter” (*n* = 204)**					**0.86**
Somewhat or strongly agree	21 (75)	124 (71)	Reference category		
Neither agree nor disagree	4 (14)	27 (15)	0.7	0.1–7.9	0.80
Strongly or somewhat disagree	3 (11)	25 (14)	0.5	0.0–7.1	0.61

^1^ The univariable analysis results for all variables assessed are reported here as there were no significant variables (*p*-value <0.05) in the analyses. ^2^ Total numbers of respondents differ between independent variables, as not all respondents answered each question, and within variables, percentages do not always sum to 100% due to rounding. ^3^ The odds ratio estimates the odds of an adopter adopting a ≥AUD$99 adult cat rather than an AUD$20 adult cat. ^4^ Bolded values are overall likelihood ratio test *p*-values for the variable; non-bolded values are Wald *p*-values for the specific category, relative to the reference category. ^5^ Categories combined for analysis as sparse or zero cells did not allow analysis of the categories separately; indicated by a common vertical line showing the categories that were pooled for analysis. ^6^ Exact logistic regression results reported (not adjusted for clustering by respondent), as random-effects logistic regression was not possible, due to sparse category combinations.

**Table S4:** Distributions of adoption outcomes by type of cat adopted (adult cat or kitten) for 266 cats adopted from an animal shelter in Australia in 2013 for variables with *p*-values ≥0.05 on univariable analyses comparing distributions between cat age groups.

Dependent Variable and Categories	Adult Cats *n* (%) ^2^	Kittens *n* (%) ^2^	Odds Ratio/Relative Risk Ratio ^3^	95% Confidence Interval ^3^	*p*-Value ^4^
**Cat retention (*n* = 266) ^5^**					**0.38**
Yes	157 (92) ^6^	90 (95)	Reference category		
No	14 (8)	5 (5)	1.6	0.6–4.6	0.38
Self-rated attachment to the adopted cat (*n* = 257) ^6^			1.5	0.7 to 3.4	0.33
Very attached	143 (86)	82 (90)			
Moderately attached	19 (12)	9 (10)			
Not at all attached	4 (2)	0			
**Agreement with the statement “I like cats” (*n* = 264) ^7^**					**0.14**
Somewhat or strongly agree	159 (94)	87 (93)	Base category		
Neither agree nor disagree	4 (2)	6 (6)	0.4	0.1–1.3	0.13
Somewhat or strongly disagree	7 (4)	1 (1)	3.8	0.4–31.8	0.21
**Would adopter choose to adopt from the shelter again in the future (*n* = 246)** ^6^			1.1	0.3–3.8	**0.87**
Yes	151 (95)	83 (95)			
Unsure	5 (3)	3 (4)			
No	3 (2)	1 (1)			
**Did the adopter intend to keep the cat? (*n* = 229)** ^8^					**0.43**
Yes	143 (98)	83 (100)	Reference category		
Unsure	3 (2)	0	2.2	0.3–∞ ^9^	0.43
**Frequency of the adopter holding/stroking/cuddling the cat/kitten (*n* = 258)** ^5^					**0.48**
Once a day or more frequently	163 (98)	90 (99)	Reference category		
Less often than once a day	4 (2)	1 (1)	2.2	0.2–20.3	0.48
**Frequency of cat/kitten being allowed inside (*n* = 259)** ^6^			0.9	0.5–1.4	**0.51**
Whenever he/she wants or always inside	144 (86)	81 (88)			
Daily	22 (13)	9 (1)			
Less often than daily	1 (1)	2 (2)			
**Indoor/outdoor status of the cat (*n* = 258)** ^6^			1.2	0.7–1.9	**0.48**
The cat/kitten is confined inside your house/apartment/unit during the day and night	58 (35)	34 (37)			
The cat/kitten is allowed to go outside during the day, but is confined to your property all of the time (e.g., in a cat enclosure or contained outdoor area) and you confine the cat/kitten inside your house/apartment/unit during the night) or the cat/kitten is allowed to go outside during the day and night but is confined to your property all of the time (e.g., in a cat enclosure or contained outdoor area)	37 (22)	22 (24)			
The cat/kitten is allowed to go outside during the day and is able to leave your property but is confined inside your house/apartment /unit during the night	51 (31)	26 (29)			
The cat/kitten is allowed to go outside during the day and night and is able to leave your property	21 (13)	9 (10)			
**A collar has been put on the cat/kitten (*n* = 258)** ^5^					**0.67**
Yes	119 (71)	68 (75)	Reference category		
No	48 (29)	23 (25)	1.5	0.2–10.5	0.67
**External identification has been put on the cat/kitten (*i.e.*, a tag with address details) (*n* = 257)** ^5^					**0.90**
Yes	91 (55)	50 (56)	Reference category		
No	76 (46)	40 (44)	1.2	0.1–9.9	0.90
**The adopter checked to make sure that the registered microchip details were correct (*n* = 244)** ^5^					**0.14**
Yes	102 (64)	44 (52)	Reference category		
No	58 (36)	40 (48)	5.3	0.6–48.3	0.14
**Did the adopter intend to take the cat to the vet yearly? (*n* = 258)** ^7^					**0.15**
Yes	146 (87)	83 (91)	Base category		
No	7 (4)	6 (7)	0.7	0.2–2.1	0.48
Not sure	14 (8)	2 (2)	4.0	0.9–18.0	0.07
**Frequency of flea control administration (*n* = 253)** ^5^					**0.52**
Every 3 months or more often	91 (56)	53 (60)	Reference category		
Less often than once every 3 months	73 (45)	36 (45)	1.3	0.5–3.2	0.41
**Frequency of de-worming medication administration (*n* = 247)** ^5^					**0.83**
Every 3 months or more often	126 (80)	72 (81)	Reference category		
Less often than once every 3 months	21 (20)	17 (19)	1.1	0.4–2.9	0.84

^1^ Variables with an overall *p*-value ≥0.05 on univariable analysis and those with an overall *p*-value <0.05 on univariable analysis are reported in Table 2. ^2^ Total numbers of respondents differ between variables, as not all respondents answered each question, and within variables, percentages do not always sum to 100% due to rounding. ^3^ Odds ratio estimates are reported for ordered logistic regression and random-effects logistic regression; these estimate the odds of any particular dependent variable (adoption outcome) category for adult cats compared to kittens. Relative risk ratio (RRR) estimates are reported for multinomial logistic regression analyses; these estimate the probability of the specified dependent variable (adoption outcome) category rather than the base outcome for adult cats compared to kittens. ^4^ Bolded values are overall likelihood ratio test *p*-values for the variable; non-bolded values are Wald *p*-values for the specific level, relative to the reference category. ^5^ Results from random-effects logistic regression as 2 categories for the dependent variable (adoption outcome). ^6^ Results from ordered logistic regression as >2 categories for the dependent variable (adoption outcome), and there was no evidence that odds are not proportional. ^7^ Results from multinomial logistic regression are reported as there was evidence that odds were not proportional. ^8^ Exact logistic regression results reported (not adjusted for clustering by respondent), as random-effects logistic regression was not possible due to sparse category combinations. ^9^ ∞ = infinity.

**Table S5:** Distributions of adoption outcomes by cat adoption price for 152 adult cats adopted from an animal shelter in Australia in 2013 for variables with overall *p*-values ≥0.05 on univariable analyses comparing distributions between adoption price groups.

Dependent Variable and Categories	≥AUD$99 Adult Cats *n* (%)^2^	AUD$20 Adult Cats *n* (%)^2^	Odds Ratio ^3^	95% Confidence Interval	*p*-Value ^4^
**Cat retention (*n* = 157)** ^5^					**0.79**
Yes	17 (90)	126 (91)	Reference category		
No	2 (11)	12 (9)	1.6	0.1–43.9	0.79
**Self-rated attachment to the adopted cat (*n* = 152)** ^6^			0.7	0.2–3.2	**0.64**
Very attached	16 (89)	114 (85)			
Moderately attached	2 (11)	16 (12)			
Not at all attached	0	4 93)			
**Satisfaction with the adopted cat (*n* = 153)** ^6^			0.7	0.2–2.4	**0.53**
Very satisfied	15 (83)	105 (78)			
Satisfied	3 (17)	23 (17)			
Neither satisfied or dissatisfied	0	3 (2)			
Dissatisfied ^9^	0	4 (3)			
**Agreement with the statement “I like cats” (*n* = 156)** ^6^			1.8	0.3–9.3	**0.51**
Somewhat or strongly agree	17 (90)	128 (93)			
Neither agree nor disagree	0	4 (3)			
Somewhat or strongly disagree	2 (11)	5 (4)			
**Would adopter choose to adopt from the shelter again in the future (*n* = 146)** ^6^			1.1	0.1–9.5	**0.90**
Yes	15 (94)	123 (95)			
Unsure	1 (6)	4 (3)			
No	0	3 (2)			
**Amount of money the adopter planned to spend on purchasing/adopting a cat from the shelter in the future (*n* = 146)** ^6^			0.5	0.3–1.2	**0.13**
≤$201	3 (19)	23 (18)			
$101–200	9 (56)	45 (35)			
$21–100	4 (25)	61 (47)			
≥$20	0	1 (1)			
**Did the adopter intend to keep the cat? (*n* = 133)** ^7^					**0.64**
Yes	14 (100)	116 (98)	Reference category		
Unsure	0	3 (3)	2.2	0.0–15.1	0.64
**Frequency of the adopter holding/stroking/cuddling the cat/kitten (*n* = 153)** ^7^					**0.70**
Once a day or more frequently	18 (100)	131 (97)	Reference category		
Less often than once a day	0	4 (3)	1.4	0.0–8.6	0.70
**Frequency of cat/kitten being allowed inside (*n* = 153)** ^6^			1.1	0.4–2.9	**0.86**
Whenever he/she wants or always inside	6 (33)	46 (34)			
Daily	9 (50)	70 (52)			
Less often than daily	3 (17)	19 (14)			
**Indoor/outdoor status of the cat (*n* = 153)** ^6^			1.7	0.7–4.2	**0.26**
The cat/kitten is confined inside your house/apartment/unit during the day and night	5 (28)	50 (37)			
The cat/kitten is allowed to go outside during the day, but is confined to your property all of the time (e.g., in a cat enclosure or contained outdoor area) and you confine the cat/kitten inside your house/apartment/unit during the night) or the cat/kitten is allowed to go outside during the day and night but is confined to your property all of the time (e.g., in a cat enclosure or contained outdoor area)	3 (17)	32 (24)			
The cat/kitten is allowed to go outside during the day and is able to leave your property but is confined inside your house/apartment /unit during the night	7 (39)	37 (27)			
The cat/kitten is allowed to go outside during the day and night and is able to leave your property	3 (17)	16 (12)			
**A collar has been put on the cat/kitten (*n*** = **153)** **^5^**					**0.05**
Yes	9 (50)	100 (74)	Reference category		
No	9 (50)	35 (26)	100.4	1.0–10,499.8	0.05
**External identification has been put on the cat/kitten (*i.e.*, a tag with address details) (*n*** = **153)** **^6^**					**0.17**
Yes	13 (72)	72 (53)	Reference category		
No	5 (28)	63 (47)	0.4	0.1–1.3	
**The adopter checked to make sure that the registered microchip details were correct (*n*** = **146) ^5^**					**0.90**
Yes	8 (50)	85 (65)	Reference category		
No	8 (50)	46 (35)	1.4	0.0–160.2	0.90
**Did the adopter intend to take the cat to the vet yearly? (*n*** = **147) ^5^**					**0.76**
Yes	16 (89)	118 (92)	Reference category		
No	2 (11)	11 (9)	1.7	0.1–46.1	
**Frequency of flea control administration (*n*** = **150)** **^5^**					**0.83**
Every 3 months or more often	10 (56)	70 (53)	Reference category		
Less often than once every 3 months	8 (44)	62 (47)	0.9	0.3–2.9	0.84
**Frequency of de-worming medication administration (*n*** = **144)** **^5^**					**0.11**
Every 3 months or more often	11 (65)	104 (82)	Reference category		
Less often than once every 3 months	6 (35)	23 (18)	2.5	0.8–7.4	0.11

^1^ All variables assessed are reported here; all had an overall *p*-value ≥0.05 on univariable analysis. ^2^ Total numbers of respondents differ between variables, as not all respondents answered each question, and within variables, percentages do not always sum to 100% due to rounding. ^3^ Odds ratio estimates are reported for ordered logistic regression and random-effects logistic regression; these estimate the odds of any particular dependent variable (adoption outcome) category for AUD$99 adult cats compared to AUD$20 adult cats. ^4^ Bolded values are overall likelihood ratio test *p*-values for the variable; non-bolded values are Wald *p*-values for the specific level, relative to the reference category. ^5^ Results from random-effects logistic regression as 2 categories for the dependent variable (adoption outcome). ^6^ Results from ordered logistic regression as >2 categories for the dependent variable (adoption outcome), and there was no evidence that odds are not proportional. ^7^ Exact logistic regression results reported (not adjusted for clustering by respondent), as random-effects logistic regression was not possible due to sparse category combinations.
